# Biological and Molecular Characterization of a Jumbo Bacteriophage Infecting Plant Pathogenic *Ralstonia solanacearum* Species Complex Strains

**DOI:** 10.3389/fmicb.2021.741600

**Published:** 2021-09-27

**Authors:** Abdelmonim Ali Ahmad, Hardian Susilo Addy, Qi Huang

**Affiliations:** ^1^Floral and Nursery Plants Research Unit, United States National Arboretum, United States Department of Agriculture-Agricultural Research Service, Beltsville, MD, United States; ^2^Department of Plant Pathology, Faculty of Agriculture, Minia University, El-Minia, Egypt; ^3^Department of Plant Protection, Faculty of Agriculture, University of Jember, Jember, Indonesia

**Keywords:** *Ralstonia solanacearum* species complex, *Ralstonia* phage, isolation, characterization, *Myoviridae*, USA, jumbo phage

## Abstract

A jumbo phage infecting *Ralstonia solanacearum* species complex strains, designated RsoM2USA, was isolated from soil of a tomato field in Florida, United States, and belongs to the family *Myoviridae*. The phage has a long latent period of 270 min and completed its infection cycle in 360 min with a burst size of approximately 32 particles per cell. With a genome size of 343,806 bp, phage RsoM2USA is the largest *Ralstonia*-infecting phage sequenced and reported to date. Out of the 486 ORFs annotated for RsoM2USA, only 80 could be assigned putative functions in replication, transcription, translation including 44 tRNAs, and structure with the main structural proteins experimentally confirmed. Phylogenetic analyses placed RsoM2USA in the same clade as Xanthomonas phage XacN1, prompting a proposal of a new genus for the two jumbo phages. Jumbo phage RsoM2USA is a lytic phage and has a wide host range, infecting each of the three newly established *Ralstonia* species: *R. solanacearum*, *R. pseudosolanacearum*, and *R. syzygii*, and significantly reduced the virulence of its susceptible *R. solanacearum* strain RUN302 in tomato plants, suggesting that this jumbo phage has the potential to be developed into an effective control against diseases caused by *R. solanacearum* species complex strains.

## Introduction

*Ralstonia solanacearum* species complex (*Rssc*) strains are soilborne vascular bacterial plant pathogens and have recently been split into three different *Ralstonia* species: *R. solanacearum*, *R. pseudosolanacearum*, and *R. syzygii* (Safni et al., 2014). They cause bacterial wilt in over 44 plant families and are a major limiting factor in the production of many economically important crops including tomato, potato, and tobacco around the world (Hayward, 1991). *Rssc* strains normally enter a host plant from soil through wounds in plant roots. They then multiply in the xylem of the plant and move through the vascular system (Vasse et al., 1995). Disease symptoms include wilting, yellowing and stunting. *Rssc* strains can be spread in soil, water, or through latently infected plant materials. Diseases caused by *Rssc* are difficult to control because of their wide host range, long survival in soil, and lack of plant resistance. As a result, alternative control measures for *Rssc* including the use of *Ralstonia*-infecting phages are being explored (Álvarez and Biosca, 2017; Buttimer et al., 2017; Wei et al., 2017; Elhalag et al., 2018; Álvarez et al., 2019). Phages are natural predators of bacteria and are not toxic to animals, humans, plants and non-target bacteria, so they are environmentally friendly. Phage application to soil pathogens like *Rssc* in humid tropical environments could minimize phage exposure to desiccation and UV light, increasing the likelihood for their success in biocontrol.

Tailed phages represent the most numerous, most widespread and probably the oldest group of bacteriophages (Hendrix, 1999). These phages have a double stranded DNA and belong to the order of *Caudovirales* under the three families of *Myoviridae*, *Podoviridae*, and *Siphoviridae*. Phages with genome sizes more than 200 kb are classified as jumbo phages (Hendrix, 2009). Due to their large sizes that limit their discovery using standard phage isolation methods (Yuan and Gao, 2017), only 108 jumbo phages had been described and sequenced before 2018, with 97 of them in the family of *Myoviridae* (Danis-Wlodarczyk et al., 2018). Most recently, with the use of metagenomics, hundreds more jumbo phage genomes have been found from diverse ecosystems with 35 genomes manually curated to completion (Al-Shayeb et al., 2020; Iyer et al., 2021). So far, 29 jumbo phages with genome sizes of more than 300 kbp have been characterized and fully sequenced. Some of them were found recently to form a new phylogenetic clade, termed “Rak2-like phages” (Abbasifar et al., 2014), while some of the other jumbo phages are distantly related to the Rak2-like jumbo phages (Attai et al., 2018; Yoshikawa et al., 2018). Two of the jumbo phages, *Agrobacterium virus Atuph07* (Attai et al., 2018) and Xanthomonas phage XacN1 (Yoshikawa et al., 2018), infect plant pathogenic bacteria including *Agrobacterium tumefaciens*, the causal agent of crown gall disease of many economically important crops, and *Xanthomonas citri*, a causal agent of devastating Asian citrus canker disease.

Compare to small-genome phages, jumbo phages have bigger icosahedral virions (head of 90–160 nm, and tail of 65–453 nm) and longer genomes (202,585–735,411 bp) that code for many more genes but with less modular genome structures (Yuan and Gao, 2017; Danis-Wlodarczyk et al., 2018; Al-Shayeb et al., 2020). They also in general have broad host ranges, so are expected to be useful as biocontrol agents. Among themselves, jumbo phages have low genome similarity and contain large numbers of proteins with unknown functions (Yuan and Gao, 2017). Recently, it has been discovered that jumbo phages including the Serratia phage PCH45 and *Pseudomonas* phages of the *Phikzlikevirus* genus form nucleus-like structures surrounded by a shell of phage proteins and centered by a phage-encoded bipolar tubulin-based spindle (PhuZ) during infection within bacterial hosts to evade CRISPR-cas immune systems, although the tubulin and shell protein gene homologs in the *Pseudomonas* and *Serratia* phages shared little similarity (Chaikeeratisak et al., 2017a,b; Malone et al., 2020).

So far, five *Ralstonia*-infecting jumbo phages have been characterized and sequenced, including *Ralstonia* phages RP12 (279,845 bp), RP31 (276,958 bp) (Matsui et al., 2017), and RSL2 (223,932 bp) (Bhunchoth et al., 2016) isolated from soil in Thailand, as well as RSL1 (231,255 bp) (Yamada et al., 2007, 2010) and RSF1 (222,888 bp) in Japan (Bhunchoth et al., 2016), all with genomes above 200 kb but less than 300 kb. In addition, a wide range of *Ralstonia* phages with genome sizes less than 200 kb have been characterized including myoviruses (Tanaka et al., 1990; Yamada et al., 2007; Fujiwara et al., 2008; Mihara et al., 2016; Askora et al., 2017; Addy et al., 2019; Kawasaki et al., 2021), podoviruses (Kawasaki et al., 2009, 2016; Bhunchoth et al., 2015; Addy et al., 2018; Ahmad et al., 2018; Álvarez et al., 2019), siphoviruses (Kawasaki et al., 2016), and inoviruses (Yamada et al., 2007; Murugaiyan et al., 2011; Van et al., 2014; Ahmad et al., 2017).

In this study, we report the discovery and characterization of a jumbo phage with a genome of more than 343 kb in size isolated from soil in the United States that specifically infects pathogenic *R. solanacearum*, *R. pseudosolanacearum*, and *syzygii* strains. We characterized the morphology, sequenced and annotated the genome of the phage. We also determined its phylogenetic relationships to other *Ralstonia* and non-*Ralstonia* jumbo phages, as well as its effect on the virulence of its susceptible *R. solanacearum* strain RUN302. These studies are important steps toward a better understanding of the jumbo phage for future exploration of its potential as a biocontrol against diseases caused by *Rssc* strains.

## Materials and Methods

### *Ralstonia solanacearum* Species Complex Strains

*Rssc* and non-*Ralstonia* strains used in this study are listed in Table 1. *R. solanacearum* strains RUN302 and UW551 were used as hosts for propagation of phage RsoM2USA. The bacterium was grown overnight in casamino acid peptone glucose (CPG) medium (Hendrick and Sequeira, 1984) at 28°C from a single colony streaked from a frozen stock, and its inoculum prepared in sterile water using OD_600_ as an initial measurement of cell density, followed by 10-fold serial dilution plating to confirm final inoculum concentration (Ahmad et al., 2017).

**TABLE 1:** Susceptibility of *R. solanacearum* species complex strains to *Ralstonia* jumbo phage RsoM2USA.

***R. solanacearum* species complex**	**Strain**	**Biovar, phylotype-sequevar**	**Origin**	Susceptibility to phage RsoM2USA^*^
*R. solanacearum*	K60	1, IIA-7	United States	S
	RUN074	1, IIB-3	Philippines	S
	RUN302	1, IIB-4	Brazil	S
	RUN651	1, IIB-4	France	S
	4153	2, II	United Kingdom	S
	Pss1475	2, II	Taiwan	S
	RUN035	2, IIB-1	Netherland	S
	UW276	2, II	Mexico	S
	UW425	2, II	Australia	S
	UW551	2, IIB-1	Kenya	S
	UW349	2T, IIB-27	Brazil	S
*R. pseudosolanacearum*	GMI1000	3, I-18	French Guiana	S
	Pss4	3, I-15	Taiwan	R
	Rs121	3, I	United States	R
	Ps191	4, I	Taiwan	S
*R. syzygii*	RUN083	2T, IV-10	Indonesia	S
**Outgroup bacteria**				
*Xanthomonas campestris* pv. *campestris strain 6*				R
*X. campestris* pv. *campestris strain 7*				R
*Pseudomonas syringae* pv. *syringae*				R

^*^Susceptibility of R. solanacearum species complex strains to phage RsoM2USA is shown as resistant (R) when no plagues were observed or susceptible (S) when clear plagues were observed.

### Phage Isolation and Purification

A pure phage designated RsoM2USA was isolated from soil obtained from a tomato field infested by *R. solanacearum* strains in Florida, United States, using *R. solanacearum* strain RUN302 as a bacterial host and the triple phage purification process described by Addy et al. (2019). The only difference is that CPG containing 0.35%, not 0.45%, agar was used as the top layer for the plaque assay to facilitate the isolation of the jumbo phage. The pure phage stock of RsoM2USA was also made, stored, and its titer determined using the method of Addy et al. (2019).

### Electron Microscopy

To characterize the morphology of the jumbo phage, phage RsoM2USA particles were treated with an equal volume of chloroform and centrifuged at 9,391 × *g* for 10 min at 4°C. The upper layer containing the phage particles was transferred into a new tube and purified by ultracentrifugation at 109,000 × *g* through a 30% sucrose cushion for 2 h at 10°C. The phage pellet was dissolved in 500 μl of SM buffer containing 50 mM Tris/HCl at pH 7.5, 100 mM NaCl, 10 mM MgSO4, and 0.01% gelatin, and used for negative staining with sodium phosphotungstate (Dykstra, 1993) before observation under a Hitachi HT7700 transmission electron microscope. At least 10 phage particles were used to estimate the phage’s morphometrics using the open source image processing program ImageJ 1.50i (Abramoff et al., 2004).

### One-Step Growth Experiment

To determine the infection cycle of phage RsoM2USA, one-step growth experiment was performed based on Ellis and Delbruck (1939) with modifications. Two hundred microliters of the overnight culture of *R. solanacearum* strain RUN302 was transferred into 9.8 ml of CPG broth and grown at 28°C with shaking until the culture reached the OD_600_ of 0.05 (5 × 10^7^ CFU/ml). Phage RsoM2USA was then added at a MOI of 1 and allowed to adsorb for 15 min at 28°C. Any non-absorbed phage particles were removed by centrifugation, followed by washing with 10 ml of CPG and centrifugation again at 6,000 × *g* for 5 min at 4°C. The pellet was resuspended in 10 ml of CPG, diluted 10,000-fold, and incubated at 28°C without shaking. An aliquot of 500 μl was taken every 30 min for 7.5 h, filtered through 0.45 μm membrane, diluted, and subjected to the plaque assay described by Addy et al. (2019) using RUN302 as a host to estimate phage titers. There were three replicates for each time point, and the experiment was repeated three times.

### Phage Host Range Determination

To determine the host specificity of the jumbo phage RsoM2USA, the purified phage was subjected to the spot test (Ahmad et al., 2017) using 16 *Rssc* and three outgroup bacterial strains (Table 1). Briefly, a double-layered CPG plate was made first by pouring a top layer containing a mixture of 3.5 ml of CPG, 0.35% agar and 250 μl of each *Rssc* strain (OD_600_ of 0.1) on top of a solidified CPG plate containing 1.5% agar. After the top layer was hardened for 15 min, 3 μl of each of a serial dilution (10^0^–10^–6^) of the phage RsoM2USA suspension (10^8^ PFU/ml) was spotted on top of the double-layer CPG plate, and incubated overnight at 28°C. The formation of plaques (lysis zones) on the plate indicated that the bacterial strain was susceptible to the phage.

### Thermal Stability Test

To find out the lethal temperature and to determine the effect of temperature on the stability of phage RsoM2USA, a thermal stability test was conducted by incubating the phage at temperatures ranging from 4°C to 90°C as described by Addy et al. (2019). Briefly, the purified phage was diluted to 1 × 10^8^ PFU/ml in SM buffer, followed by incubation of 1 ml of the diluted phage suspension at each of the designated temperatures for 1 h. To estimate phage numbers after incubation, the phage suspension was serially diluted in SM buffer and subjected to plaque assay using *R. solanacearum* RUN302 as a host. There were three replicates for each temperature and the experiment was repeated once.

### Phage DNA Extraction, Sequencing, and Sequence Analysis

Phage DNA was extracted from purified phage particles using either a phenol-chloroform method (Sambrook and Russell, 2001) or the Phage DNA Isolation kit (Norgen Biotek Corp, Canada). The phage DNA was sequenced on an Illumina MiSeq with 2 × 150 bp reads, and the genome sequence assembled using Spades v3.11 commercially by SeqMatic (Fremont, California). Potential open reading frames (ORFs) larger than 50 amino acids (aa), and putative tRNAs in phage RsoM2USA were identified using PHASTER (Arndt et al., 2016), GeneMarkS (Besemer, 2001), DNASTAR (DNASTAR Inc., United States), and tRNAscan-SE 2.0^1^ (Chan and Lowe, 2019). Homology searches for each identified ORF were performed using BLAST/PSI-BLAST against NCBI’s protein databases. An *e*-value threshold of e–4 or less was used for two proteins to be considered a match. Functional annotation and pathway identification were done using KEGG Orthology (KO) (Kanehisa et al., 2016) and Balst2go (Götz et al., 2008). Codon usage frequencies in the jumbo phage RsoM2USA and *R. solanacearum* strain RUN302 were calculated using the Codon Usage program available at https://www.bioinformatics.org/sms2/codon_usage.html. Complete genome sequences of 47 jumbo phages in the family of *Myoviridae* with a genome size more than 200 kb were downloaded from GenBank (Supplementary Table 1) and their genome sequences were compared using a dotplot generated in Gepard ver. 1.40 by calculating the similarity of the genome sequences and displaying similar DNA fragments with default parameters (word length of 10) (Krumsiek et al., 2007). The Average Nucleotide Identity (ANI) value was calculated using OrthoANI Tool version 0.93.1 to measure the overall similarity between genome sequences (Lee et al., 2016) and the heatmap was generated by choosing Color Scales for the conditional formatting in Microsoft Excel. For phylogenetic analysis, amino acid sequences of the major phage capsid protein, terminase large subunit protein, and portal vertex protein were first aligned using MUSCLE (MUltiple Sequence Comparison by Log-Expectation), followed by construction of phylogenetic trees using the Maximum Likelihood method implemented in MEGA-X (Kumar et al., 2018) version 10.0.5^2^ with 1,000 bootstrap replications. The terminase large subunit protein tree was built for 25 jumbo phages, while the major capsid or portal vertex protein tree was constructed for 24 and 19 phages including RsoM2USA, respectively. This is because annotations for the major capsid protein in Klebsiella phage K64-1, and for the portal vertex protein in Edwardsiella phage pEtSU, Prochlorococcus phage PSSM2, and *Ralstonia* phages RSF1, RSL2, RP12, and RP31 were not found (Supplementary Table 2).

### Identification of Phage Virion Proteins

Purified phage particles (5 × 10^10^) were denatured by mixing with 4 × Laemmli sample buffer and heating at 95°C for 5 min. After cooling down on ice, 30 μl of the sample were subjected to SDS-polyacrylamide gel electrophoresis (PAGE) (12% wt/vol polyacrylamide) according to the methods of Laemmli (1970). Protein bands were visualized with Coomassie Brilliant Blue R250 stain reagent (Thermo Fisher Scientific, United States). The most abundant bands were excised from the gel and sent to ProtTech, Inc. (Phoenixville, Pennsylvania) for protein identification using liquid chromatography-tandem mass spectrometry (LC-MS/MS). The mass spectrometric data is used to search against NCBI’s most recent non-redundant protein database, and against each of the predicted protein sequences of RsoM2USA using ProtTech’s ProtQuest software suite.

### Virulence Assay

Tomato plants (*Lycopersicon esculentum* Mill. cv. “bonnie best”) were grown, transplanted and inoculated as described previously (Ahmad et al., 2017), except that for plant inoculation, 30 ml of *R. solanacearum* strain RUN302 (10^8^ cells/ml) was first poured into each pot. This was followed immediately by pouring either 30 ml of phage RsoM2USA suspension (10^8^ PFU/ml) for a MOI of 1, or with water as a non-phage treatment control. Negative control plants were inoculated with 60 ml of water. Inoculated plants were rated daily using a disease index (DI) of 0–4 (Roberts et al., 1988). There were five plants for each treatment and the experiment was repeated three times.

### Statistical Analysis

Means of disease index between untreated (wild type) and jumbo phage RsoM2USA-treated *R. solanacearum* RUN302 strains were analyzed for significant differences using the *t*-test in Microsoft Excel.

### Genome and Protein Sequence Accession Numbers

The complete genome sequence of Ralstonia phage RsoM2USA was deposited to GenBank under the accession number MG752970 (Supplementary Table 1). The accession numbers for the genome and megaplasmid sequences of *R. solanacearum* strain IBSBF1503 (alternative ID of RUN302) are CP012943.1 and CP012944.1, respectively.

## Results and Discussion

### Isolation and Morphological Characterization of *Ralstonia* Phage RsoM2USA

**FIGURE 1:**
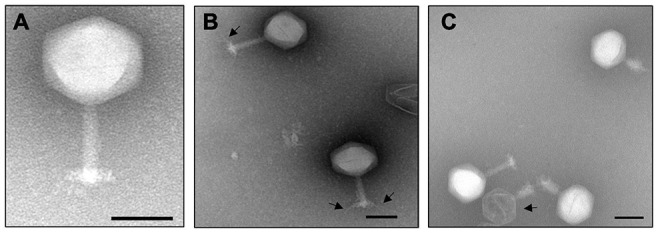
Transmission electron micrograph of the purified jumbo phage RsoM2USA virions **(A)**, as well as virions with tail fibers **(B)** and an empty virion **(C)** as indicated by arrows. The scale represents 100 nm.

A phage was isolated from a soil sample collected from a *Rssc*-infested tomato field in Florida, United States. The phage produced small and clear plaques with a diameter of approximately 1–2 mm on the top layer containing 0.35% agar of a double layered CPG plate using *Rssc* strain RUN302 as a host. The phage has an icosahedral head of 142 ± 7 nm (*n* = 10) in diameter, and a long tail with a length of 125 ± 5 nm (*n* = 10) (Figure 1). The phage also has a baseplate and tail fibers of approximately 70 nm in length (Figure 1B). Since the morphology of the phage is typical for members of the family *Myoviridae*, the phage was designated *Ralstonia* phage RsoM2USA by using our systematic phage naming approach (Ahmad et al., 2018), since it is the second *Rssc* strain-infecting phage belonging to the family *Myoviridae* that was isolated from the United States after *Ralstonia* phage RsoM1USA (Addy et al., 2019). Compared to RsoM2USA, phage RsoM1USA has a much smaller icosahedral head of 63 nm × 66 nm but a longer contractile tail of 152 nm in size (Addy et al., 2019).

### Host Range, Infection Cycle and Thermal Stability of *Ralstonia* Phage RsoM2USA

**FIGURE 2:**
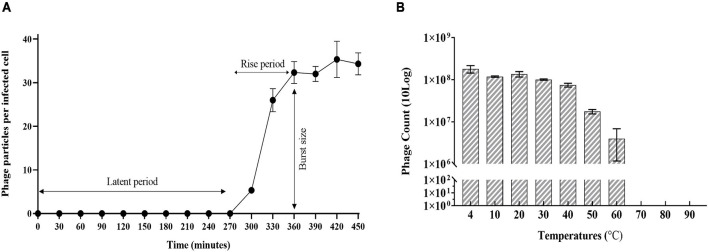
Growth characteristics of *Ralstonia* jumbo phage RsoM2USA. **(A)** One-step growth curve of the phage with *R. solanacearum* strain RUN302 as the host. The phage was added at a MOI of 1 and allowed to adsorb for 15 min at 28°C. Phage titers were determined every 30 min using the plaque assay. The latent period is when no release of phage particles was observed. The rise period begins with the end of the latent period and ends when the increase in phage titers ceases. The burst size is the average number of phage particles released per infected cell. **(B)** Effect of temperature on the stability of phage RsoM2USA. 10^8^ PFU of phage RsoM2USA was incubated at each temperature point and the number of phage was estimated by plaque assay using *R. solanacearum* RUN302 as a host 1 h after incubation. Means are based on three separate experiments, each containing three replicates. Bars indicate standard deviations.

Sixteen *Rssc* strains originally isolated from different geographic regions of the world belonging to different biovars/phylotypes/sequevars were tested for their susceptibility to *Ralstonia* phage RsoM2USA. Phage RsoM2USA infected 14 of the 16 tested *Rssc* strains in all tested biovars (1, 2, 2T, 3, and 4) and in all the three different *Ralstonia* species—*R. solanacearum*, *R. pseudosolanacearum*, and *R. syzygii* strains originated from different countries, indicating a wide host range of the phage (Table 1). The phage, however, did not infect tested *Xanthomonas campestris* and *Pseudomonas syringae* strains, indicating its specificity to the three *Ralstonia* species (Table 1). RsoM2USA has a lytic infection cycle, which was determined to be 360 min, with a latent period of 270 min, the longest latent period determined so far for jumbo phages (Monson et al., 2011; Abbasifar et al., 2014; Yoshikawa et al., 2018), followed by a 90-min rise period with a burst size of 32 ± 3 particles per infected cell (Figure 2A). A similar burst size of approximately 30 was found for jumbo phage XacN1, although its latent period was determined to be 90 min and growth cycle completed within 240 min (Yoshikawa et al., 2018). The phage was stable from 4 to 40°C, since no significant difference in its titer was found under this temperature range (Figure 2B). Significant loss in the phage titer, however, was observed at 50 and 60°C, and no phage particles were detected after the phage was incubated at 70, 80, and 90°C for 1 h, suggesting that the lethal temperature for the phage is approximately 70°C (Figure 2B).

### General Genomic Features of *Ralstonia* Phage RsoM2USA

The complete genome of the Ralstonia phage RsoM2USA was determined to be 343,806 bp in size (GenBank accession no. MG752970), resulting in the classification of RsoM2USA as a jumbo phage. Such genome size also makes RsoM2USA the largest *Ralstonia* phage sequenced and reported so far, since it is larger than previously reported jumbo *Ralstonia* phages RSF1, RSL1, RSL2, RP31, and RP12 with their genome sizes ranging from 222,888 to 279,845 bp (Yamada et al., 2007, 2010; Bhunchoth et al., 2016; Matsui et al., 2017). RsoM2USA also represents the third largest phage infecting plant pathogenic bacteria after *Agrobacterium virus Atuph007* and Xanthomonas phage XacN1, and the 23rd largest phage reported to date (Buttimer et al., 2017; Casey et al., 2017; Yuan and Gao, 2017; Attai et al., 2018; Yoshikawa et al., 2018; Al-Shayeb et al., 2020). The G + C content of the RsoM2USA genome is 41%, significantly lower than that of its *Ralstonia* host genomes (e.g., G + C content of 67% in *Rssc* strain IBSBF1503 (accession number of NZ_CP012943.1 in GenBank) and 66.97% in strain GMI1000 (NC_003295). A total of 486 potential open reading frames (ORFs) were identified (Supplementary Figure 1 and Supplementary Table 3). Among them, 239 had no significant similarity with any of the protein sequences in the searched databases, 167 were annotated as conserved, conserved hypothetical or unnamed proteins with no assigned functions, and only 80 were predicted proteins shared homology with other phages or bacteria with assigned functions based on KEGG analysis (Table 2 and Supplementary Table 3). This is similar to other jumbo phages, in which even though many more proteins were predicted from the genomes of jumbo than from smaller phages, the majority of the jumbo phages’ proteins has no matches in the current databases with undiscovered functions that prevented detailed comparisons among jumbo phages.

**TABLE 2:** List of annotated ORFs of jumbo phage RsoM2USA with predicted functions and proteins, and their BLASTp results.

**Function**	**ORF**	**Strand**	**Start nt**	**Stop nt**	**Length (aa)**	**Similarity to best functional homologs**	***E*-Value**	**Homolog accession no.**
Replication, recombination and repair	ORF28	−	21,854	23,371	506	SpoVR family protein [*Vibrio parahaemolyticus*]	0	WP_025610357
	ORF129	−	85,243	85,968	242	Homing endonuclease [Escherichia phage vB_EcoM_112]	1.00E-23	YP_009030743.1
	ORF195	−	121,703	122,092	130	DUF2493 domain-containing protein [*Bacillus pumilus*]	3.00E-25	WP_074041829.1
	ORF213	−	135,426	136,346	307	Hypothetical protein DRJ15_13365 [Bacteroidetes bacterium]	8.00E-93	RLD77522.1
	ORF234	−	149,131	150,414	428	DNA helicase-like protein [Salicola phage SCTP-2]	7.00E-87	ASV44158.1
	ORF253	+	162,753	164,330	526	Terminase large subunit [Xanthomonas phage XacN1]	7.00E-136	BBA65403.1
	ORF358	−	225,112	226,089	326	GDP-mannose 4,6-dehydratase [Enterobacter hormaechei]	1.00E-92	WP_190319761
	ORF371	+	248,317	250,059	581	Endonuclease subunit [uncultured Caudovirales phage]	2.00E-87	CAB4241474.1
	ORF435	−	307,406	308,074	223	Putative exonuclease [Serratia phage phiMAM1]	6.00E-40	YP_007349054.1
	ORF441	−	312,188	313,516	443	DNA primase-helicase subunit [Agrobacterium phage Atu_ph07]	4.00E-95	ASV44692.1
	ORF448	−	319,603	320,598	332	DNA polymerase I [Syntrophomonadaceae bacterium]	3.00E-93	AVH85376.1
	ORF451	−	321,631	324,054	808	DNA polymerase [Xanthomonas phage XacN1]	8.00E-167	BBA65491.1
	ORF458	−	326,884	327,468	195	DNA polymerase III epsilon subunit [Xanthomonas phage XacN1]	3.00E-32	BBA65371.1
Translation, ribosomal structure and biogenesis	ORF8	−	6,927	7,346	140	Cytidine and deoxycytidylate deaminase zinc-binding region [uncultured *Eubacterium* sp.]	1.00E-26	SCI18574.1
	ORF22	−	16,776	17,813	346	Bifunctional nicotinamide-nucleotide adenylyltransferase/Nudix hydroxylase [Chitinivorax tropicus]	1.00E-96	WP_184035971.1
	ORF157	−	103,724	104,122	133	Aminoacyl-tRNA hydrolase [Steroidobacter agaridevorans]	7.00E-53	WP_202623994.1
	ORF161	−	105,942	106,757	272	DUF4343 domain-containing protein [*Polaromonas* sp. CF318]	2.00E-68	WP_007869793.1
	ORF183	−	114,961	115,776	272	Putative Thg1 [Pseudomonas phage 201phi2-1]	5.00E-67	YP_001957040.1
	ORF204	−	129,120	130,403	428	ATP-dependent DNA ligase [*Bacillus* phage SP-10]	2.00E-38	YP_007003455.1
	ORF222	−	141,515	142,111	199	Alpha/beta fold hydrolase [Verrucomicrobiaceae bacterium]	1.00E-32	RYD62074.1
	ORF397	+	266,707	267,747	347	RNA ligase [Caulobacter phage Cr30]	5.00E-98	YP_009098789.1
	ORF470	−	332,504	333,007	168	Macro domain-containing protein [*Paraburkholderia* sp. UCT31]	2.00E-35	WP_187631037.1
	ORF480	−	336,962	338,041	360	2’-5’ RNA ligase [*Paraburkholderia* sp. C35]	2.00E-84	WP_109482880.1
Transcription	ORF14	−	10,780	12,177	466	Nicotinate phosphoribosyltransferase [*Variovorax paradoxus*]	0	WP_081267491.1
	ORF57	−	43,612	45,078	489	TROVE domain-containing protein [Candidatus Woesebacteria bacterium RBG_13_36_22]	5.00E-143	OGM09089.1
	ORF193	−	119,483	121,156	558	gp73 [*Bacillus virus G*]	3.00E-109	YP_009015384.1
	ORF194	−	121,156	121,710	185	Dihydrofolate reductase [*Bacillus* sp. VT-16-64]	1.00E-30	WP_077113372.1
	ORF217	−	138,548	139,012	155	CMP deaminase [*Mariniphaga anaerophila*]	1.00E-44	WP_073001946.1
	ORF248	−	159,734	160,165	144	Adenylylsulfate kinase [*Beijerinckia* sp. 28-YEA-48]	8.00E-53	SEB56588.1
	ORF446	−	317963	319,018	352	CobS [Synechococcus phage S-WAM2]	8.00E-49	YP_009324303.1
	ORF485	−	341,063	342,979	639	GyrB Type IIA topoisomerase (DNA gyrase/topo II, topoisomerase IV), B subunit [uncultured Caudovirales phage]	0.00E + 00	CAB4159554.1
Posttranslational modification, protein turnover and chaperones	ORF3	−	3,079	3,714	212	Heat-shock protein [*Microvirga* sp. BSC39]	5.00E-22	WP_036354424.1
	ORF112	−	77,678	78,244	189	NAD-dependent deacylase [*Thermococcus gorgonarius*]	1.00E-43	WP_088885183.1
	ORF223	−	142,171	142,503	111	Thioredoxin [*Crenothrix* sp. D3]	1.00E-25	OTE97860.1
Nucleotide transport and metabolism	ORF10	−	7,607	8,443	279	Putative thymidylate synthase [Xanthomonas phage Xp15]	2.00E-50	YP_239304.1
	ORF225	−	142,794	143,939	382	Ribonucleotide-diphosphate reductase subunit beta [*Shewanella colwelliana*]	3.00E-141	WP_028763880.1
	ORF226	−	144,040	146,370	777	Ribonucleoside-diphosphate reductase subunit alpha [*Comamonas* sp. B-9]	0	WP_027011154.1
	ORF473	−	334,304	335,233	310	Td thymidylate synthetase [Acinetobacter phage Acj61]	3.00E-167	YP_004009838.1
Amino acid transport and metabolism	ORF5	−	5,117	5,956	280	Nitrate reductase [*Herbaspirillum chlorophenolicum*]	6.00E-69	WP_050467745.1
	ORF477	−	336,023	336,469	149	NTP-PPase [*Caudovirales* sp. ctOwN3]	3.00E-32	QGH72159.1
	ORF262	+	169,665	170,243	193	Phospholipase D family protein [*Chlorobaculum limnaeum*]	1.00E-33	WP_069809568.1
Signal transduction mechanisms	ORF2	−	1,890	3,020	377	Beta glucosyl transferase [Enterobacter phage CC31]	5.00E-38	YP_004009897.1
	ORF17	−	13,266	13,880	205	General stress protein 16U [Pseudomonas phage VCM]	2.00E-45	YP_009222754.1
	ORF18	−	13867	14,931	355	DUF475 domain-containing protein [*Sphingomonas sanguinis*]	7.00E-98	WP_058733334.1
	ORF19	−	14,888	15,565	226	Von Willebrand factor type A domain-containing protein [Rhizobium phage RHph_TM30]	6.00E-86	QIG71336.1
	ORF30	−	24,727	26,670	648	PrkA family serine protein kinase [*Alcanivorax* sp. CP2C]	0	WP_067606528.1
	ORF35	−	28,475	29,101	209	Metallophosphoesterase [Bacillus phage Troll]	7.00E-32	YP_008430899.1
	ORF43	−	34,664	35,284	207	Putative Hef-like homing endonuclease [*Acinetobacter virus 133*]	1.00E-06	YP_004300760.1
	ORF237	−	151,481	152,338	286	Serine/threonine protein phosphatase 1 [*Methylocaldum* sp. 175]	1.00E-39	SMF95613.1
	ORF239	−	153,493	154,359	289	Single-stranded DNA binding protein [Xanthomonas phage XacN1]	4.00E-44	BBA65400.1
Cell wall/membrane/envelope biogenesis	ORF33	−	27,527	28,144	206	Restriction endonuclease [*Betaproteobacteria bacterium* RIFCSPLOWO2_12_FULL_62_13]	5.00E-55	OGA37234.1
	ORF60	−	46,489	47,295	269	Putative family 9 glycosyl transferase [Caulobacter phage CcrColossus]	5.00E-17	YP_006988365.1
	ORF155	−	101,717	103,162	482	VCBS repeat-containing protein [*Loktanella vestfoldensis*]	7.00E-28	WP_087211375.1
	ORF425	+	299,739	301,409	557	Murein DD-endopeptidase MepM [Syntrophomonadaceae bacterium]	8.00E-61	MBT9137488.1
	ORF426	+	301,412	301,699	96	PaaR repeat-containing protein [*Ruegeria mobilis*]	8.00E-10	WP_074712575.1
	ORF486	−	343,044	343,793	250	Bifunctional protein GlmU [bacterium BMS3Bbin11]	8.00E-06	GBE46073.1
Structure	ORF356	−	223,631	224,212	194	Structural protein [Xanthomonas phage XacN1]	4.00E-44	BBA65499.1
	ORF359	−	226,174	227,745	524	Tail sheath protein [Xanthomonas phage XacN1]	8.00E-94	BBA65515.1
	ORF361	−	229,048	236,670	2541	Structural protein [Pectobacterium phage CBB]	2.00E-17	AMM43801.1
	ORF362	−	236,776	240,420	1215	Baseplate wedge [Cronobacter phage vB_CsaM_GAP32]	2.00E-93	YP_006987336.1
	ORF363	−	240,586	240,963	126	Baseplate wedge [Xanthomonas phage XacN1]	2.00E-26	BBA65471.1
	ORF364	−	240,963	242,090	376	Baseplate hub subunit and tail lysozyme [Pseudomonas phage vB_PaeM_PA5oct]	2.00E-29	QCG76015.1
	ORF367	−	244,672	245,745	358	Structural protein [Serratia phage BF]	2.00E-17	AQW88767.1
	ORF375	−	253,025	253,825	267	Baseplate hub subunit [Agrobacterium phage Atu_ph07]	2.00E-23	ASV44759.1
	ORF378	+	257,102	258,826	575	Portal vertex protein [Xanthomonas phage XacN1]	4.00E-133	BBA65449.1
	ORF379	+	259,178	259,705	176	Prohead core scaffolding protein and protease [Xanthomonas phage XacN1]	1.00E-51	BBA65445.1
	ORF383	+	261286	262,521	412	Major capsid protein [Salicola phage SCTP-2]	7.00E-104	ASV44110.1
	ORF401	+	268,348	269,829	494	Neck protein [Pectobacterium phage CBB]	2.00E-23	AMM43766.1
	ORF406	+	274017	274,829	271	Proximal tail sheath stabilization [uncultured Mediterranean phage uvMED]	7.00E-22	BAR27635.1
	ORF410	+	278,320	279,018	233	Putative tail fiber protein [Pseudomonas phage Noxifer]	2.00E-07	ARV77307.1
	ORF415	+	283,386	285,101	572	Phage-related tail fiber protein [uncultured Mediterranean phage uvMED]	1.00E-29	BAR25957.1
Integral component of membrane	ORF54	−	41,514	42,440	309	Phage shock protein A [*Lewinella agarilytica*]	1.00E-22	SEQ17255.1
	ORF187	−	117,319	117,720	134	DUF3307 domain-containing protein [*Sulfitobacter* sp. 20_GPM-1509m]	4.00E-19	WP_028956004.1
Coenzyme transport and metabolism	ORF271	+	174,077	174,772	232	VWA domain-containing protein [*Blautia hydrogenotrophica*]	8.00E-49	WP_005952142.1
	ORF445	−	316,066	317,973	636	Peptidase [uncultured Mediterranean phage uvMED]	3.00E-25	BAR35475.1
Mobilome: prophages, transposons	ORF37	−	29,535	29,810	92	Hypothetical protein E4H12_13870 [Candidatus Thorarchaeota archaeon]	1.00E-13	TFG95161.1
	ORF228	−	147,097	147,360	88	MULTISPECIES: DUF4326 domain-containing protein [Thioalkalivibrio]	1.00E-31	WP_013006599.1
	ORF260	−	167,955	168,383	143	Head completion protein [Synechococcus phage ACG-2014f]	5.00E-37	AIX21328.1
tRNA	ORF84	−	59,431	60,477	349	tRNA(Ile)-lysidine synthase (tRNA(Ile)-lysidinesynthetase) (tRNA(Ile)-2-lysyl-cytidine synthase) [*Rickettsiella grylli*]	2.00E-32	EDP46981.1
	ORF156	−	103,221	103,724	168	Glutaminyl-tRNA synthase (glutamine-hydrolyzing) subunit B [Candidatus Saccharibacteria bacterium 49-20]	5.00E-08	OJU87614.1

### Comparative Genomics and Phylogenetic Relationships Between *Ralstonia* Jumbo Phage RsoM2USA and Other Jumbo Phages in the Family of *Myoviridae*

**FIGURE 3:**
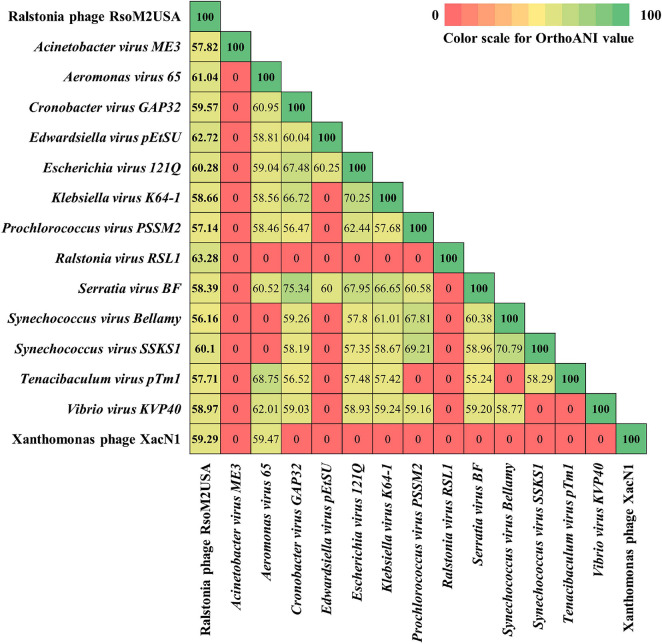
Heatmap chart generated from Orthologous Average Nucleotide Identity (OrthoANI) values of 15 phages in the family of *Myoviridae* that share similarity to *Ralstonia* phage RsoM2USA.

Based on ICTV’s 2020 release of virus taxonomy,^3^ the family of *Myoviridae* consists of 8 subfamilies (*Emmerichvirinae, Eucampyvirinae*, *Gorgonvirinae*, *Ounavirinae*, *Peduovirinae*, *Tevenvirinae*, *Twarogvirinae*, and *Vequintavirinae*) with 64 genera and 294 species (Kuhn et al., 2013). In addition, 153 genera with 331 species are classified directly under the family of *Myoviridae* for a total of 217 genera and 625 species. In addition to Xanthomonas phage XacN1 and Serratia phage PCH45, species-undefined jumbo phages in *Myoviridae*, only 44 of the genera in *Myoviridae* contain jumbo phages with genome sizes more than 200 kb (Hendrix, 2009; Supplementary Table 1). Genomic relationships between *Ralstonia* jumbo phage RsoM2USA and 46 representative jumbo phages in the family of *Myoviridae* was therefore determined using the whole genome dot plot (Supplementary Figure 2). This was done by comparing the genome sequences of RsoM2USA with Xanthomonas phage XacN1, Serratia phage PCH45, and representative phages from each of the 44 jumbo phage-containing genera of *Myoviridae*, including five previously reported *Ralstonia* jumbo phages RSF1 and RSL2 (Bhunchoth et al., 2016), RSL1 (Yamada et al., 2010), RP12 and RP31 (Matsui et al., 2017; Supplementary Figure 2). Our dot plot result indicated that RsoM2USA is a novel phage because RsoM2USA did not show similarity pattern (indicated by absence of diagonal lines) with any of the 46 phages including the five *Ralstonia* jumbo phages (Supplementary Figure 2). Further analysis using the Orthologous Average Nucleotide Identity (OrthoANI) value revealed that phage RsoM2USA shared OrthoANI values ranging from 56.16 to 63.28% to only 14 of the 46 jumbo phages including *Ralstonia virus RSL1* (*Mieseafarmvirus*) and Xanthomonas phage XacN1 (Figure 3 and Supplementary Table 4). The fact that the shared OrthoANI values of RsoM2USA with the 14 jumbo phages are less than 95% suggests that the jumbo phage RsoM2USA belongs to a new species in *Myoviridae*, since the OrthoANI value of 95% is used for demarcation of species (Lee et al., 2016).

**FIGURE 4:**
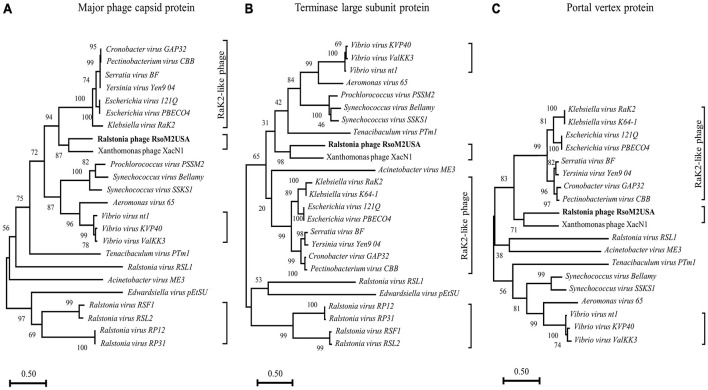
Phylogenetic relationships among *Ralstonia* jumbo phage RsoM2USA, other *Ralstonia* jumbo phages and related non-*Ralstonia* jumbo phages in *Myoviridae.* The phylogenetic trees were generated using MEGA-X (Kumar et al., 2018), based on the annotated major phage capsid protein **(A)**, terminase large subunit protein **(B)**, and portal vertex protein **(C)**. Vertical distances are arbitrary, but the horizontal branches are proportional to genetic distance, Bootstrap values (1,000 replications) are represented at the nodes of the branches. Jumbo phages grouped into different clusters are shown in each phylogenetic tree with the right-side brackets. The cluster with Rak2-like phages are labeled.

To determine evolutional relationships between RsoM2USA and other *Ralstonia* and non-*Ralstonia* jumbo phages, phylogenetic analysis was conducted for RsoM2USA, five other *Ralstonia* jumbo phages in *Myoviridae*, and 19 jumbo phages representing all species with OrthoANI values of more than 56% (Figure 4 and Supplementary Tables 2, 4). Since jumbo phages are highly divergent, no set of genes is present in all phages. Three “signature gene products” including the major capsid protein, terminase large subunit protein and portal vertex protein were used in this study, since they are conserved in many jumbo phages and generally used for phylogenetic analysis of jumbo phages (Attai et al., 2018; Yoshikawa et al., 2018). A phylogenetic tree based on the predicted amino acid sequences of major capsid proteins showed that phage RsoM2USA is more closely related to Xanthomonas phage XacN1, but not to any of the five *Ralstonia* and the other 19 non-*Ralstonia* jumbo phages (Figure 4A). The phylogenetic tree based on the predicted amino acid sequences of terminase large subunit or portal vertex proteins also consistently placed RsoM2USA in the same clade with Xanthomonas phage XacN1, not with any other *Ralstonia* and non-*Ralstonia* jumbo phages used for the comparison (Figures 4B,C). *Ralstonia virus RSF1* and *Ralstonia virus RSL2* in genus *Chiangmaivirus* were group together in the same clade, so were *Ralstonia virus RP12* and *Ralstonia virus RP31* in genus *Ripduovirus* (Figures 4A,B). The four *Ralstonia* jumbo phages in the two genera were more closely related to each other than to *Ralstonia virus RSL1* in genus *Mieseafarmvirus*, and to RsoM2USA, as revealed by phylogenetic trees based on the predicted amino acid sequences of the major capsid and terminase large subunit proteins, respectively (Figures 4A,B). Major capsid protein and terminase large subunit protein trees all support the grouping of similar phage clusters according to the relatedness of the jumbo phages (Figures 4A,B). One cluster is well-known containing Rak2-like phages previously reported by Attai et al. (2018) and Yoshikawa et al. (2018) that include *Klebsiella virus Rak2* (and K64-1 in Figure 4B) (genus *Alcyoneusvirus*), *Escherichia* phages 121Q and PBECO4 (genus *Asteriusvirus*), *Serratia virus BF* and *Yersinia virus Yen9-04* (genus *Eneladusvirus*), as well as *Cronobacter virus GAP32*, and *Pectinobacterium virus CBB* (genus *Mimasvirus*). A cluster consisting of *Vibrio* phages nt1, ValKK3, and KVP40 formed another cluster for jumbo phages belonging to the genus *Schizotequatroviru*s in the subfamily of *Tevenvirinae*. The portal vertex protein tree also supported similar grouping of phage clusters, although missing the cluster of *Ralstonia* jumbo phages in genera *Chiangmaivirus* and *Ripduovirus*, since no annotation for portal vertex protein has been found for the four *Ralstonia* jumbo phages. Interestingly and unexpectedly, RsoM2USA is grouped in the same cluster as the species-undefined Xanthomonas phage XacN1 by all three trees (Figure 4), suggesting that the two jumbo phages are distantly related to the Rak2-like phages as previously found for Xanthomonas phage XacN1 (Yoshikawa et al., 2018) and prompting a proposal of a new genus for the two jumbo phages. Comparisons of the general characteristics of the two phages and their best hit protein homologs are summarized in Supplementary Table 5.

### Functional Annotation

Eighty of the 486 ORFs were predicted to function in the following categories: replication, recombination and repair; translation, ribosomal structure and biogenesis; transcription; posttranslational modification, protein turnover, and chaperones; nucleotide transport and metabolism; amino acid transport and metabolism; signal transduction mechanisms; cell wall/membrane/envelope biogenesis; structure; integral component of membrane; coenzyme transport and metabolism; as well as mobilome: prophages and transposons (Table 2). BLASTn and manual searches for the shell protein and tubulin genes in the RsoM2USA genome yielded no orthologs to those in *Pseudomonas* and *Serratia* jumbo phages (Chaikeeratisak et al., 2017a,b; Malone et al., 2020), although orthologs were found in other *Ralstonia* jumbo phages including *Ralstonia virus RP12, Ralstonia virus RP31, Ralstonia virus RSL2*, and *Ralstonia virus RSF1*, suggesting that RsoM2USA may use a different strategy to evade CRISPR-cas DNA targeting by its bacterial host.

#### Replication, Recombination and Repair

Thirteen ORFs of RsoM2USA were predicted to encode proteins that play a role in the phage’s DNA replication, recombination and repair, including ORF129 for a homing endonuclease, ORF234 for a DNA helicase-like protein, ORF253 for a terminase large subunit, ORF371 for an endonuclease subunit, ORF435 for a putative exonuclease, ORF441 for a DNA primase-helicase subunit, ORF448 for a DNA polymerase I, ORF451 for a DNA polymerase and ORF458 for a DNA polymerase III epsilon subunit (Table 2). These ORFs had homologs in other phages including Escherichia phage vB_Ecom_112 (the phage name given here and thereafter is based on the one obtained from BLASTp result without updating to its most recent taxonomy name for simplicity) (ORF129), Xanthomonas phage XacN1 (ORFs 253, 451, and 458), Salicola phage SCTP-2 (ORF234), Serratia phage phiMAM1 (ORF435), and Agrobacterium phage Atu_ph07 (ORF441), as well as in bacterium *Bacillus pumilus* (ORF195). Like *Agrobacterium tumefaciens* jumbo phage Atu_pho7 (Attai et al., 2018), *Ralstonia* jumbo phage RsoM2USA was found to encode predicted DNA polymerase (ORF451), and DNA polymerase III epsilion subunit (ORF458), suggesting that the polymerases may contribute to both DNA replication and 3′-5′ exonuclease activity. RsoM2USA was also predicted to encode a SpoVR family protein (ORF28), with 56% amino acid sequence identity to its counterpart in *Vibrio parahaemolyticus*, which may be involved in cell cycle control, cell division, and chromosome partitioning based on KEGG pathway and Blast2GO analyses (Table 2 and Supplementary Table 3).

#### Nucleotide Transport and Metabolism

The genome of RsoM2USA encodes four proteins that are predicted to contribute to nucleotide transport and metabolism (Table 2). These include both alpha and beta subunits of ribonucleotide-diphosphate reductase (RNR) (ORFs 225 and 226) which may contribute to the oxidoreductase process by catalyzing the reductive synthesis of deoxyribonucleotides from ribonucleotides and providing the precursors necessary for DNA synthesis (Dwivedi et al., 2013; Sengupta, 2014). In addition, ORFs 10, and 473 were predicted to encode a putative thymidylate synthase and a Td thymidylate synthetase, respectively (Table 2). Thymidylate synthase is a key enzyme in DNA synthesis. Td thymidylate synthetase provides the sole *de novo* pathway for production of dTMP and is the only enzyme in folate metabolism in which the 5,10-methylenetetrahydrofolate is oxidized during one-carbon transfer (Hardy et al., 1987). It is also essential for regulating the balanced supply of the 4 DNA precursors in normal DNA replication (Muralidharan et al., 2017).

#### tRNAs, Codon Usage, and tRNA Processing Genes

The 41% G + C content of the RsoM2USA genome is significantly lower than the 67% one of the bacterial host genome. Other *Ralstonia* phages including RP12 and RP31 (Matsui et al., 2017), RSF1 and RSL2 (Bhunchoth et al., 2016), RSL1 (Yamada et al., 2010), and RP13 (Kawasaki et al., 2021) all have a characteristic lower than host G + C content ranging from 39.2 to 58%. The gap in genomic nucleotide compositions between the phage and its host (thus codon usage) makes it difficult for the phage to adapt to the translation machinery of the host cell. It is common, therefore, for some phages, especially lytic ones to direct the synthesis of their own tRNAs to ensure the effective rate of translation (Bailly-Bechet et al., 2007). The genome of RsoM2USA was annotated to encode 44 tRNA genes, including 43 canonical tRNAs corresponding to all amino acids except isoleucine (Ile) (Supplementary Table 6). The remaining tRNA is a suppressor with an anticodon of CUA, suggesting a reading through of the UAG (amber) stop codon. The UAG stop codon is abundant in both the phage RsoM2USA (*n* = 1,630) and *R. solanacearum* (*n* = 6,425) genomes, suggesting that the suppressor tRNA is targeted by several potential genes. Except the suppressor tRNA, all the tRNAs encoded in the RsoM2USA genome were also found in the bacterial host genome, suggesting that the tRNAs of RsoM2USA did not improve its translation capacity. The fact that some of the phage tRNAs correspond to codons that are more frequently used in the phage genome (Supplementary Table 6) suggested that the phage may modulate the concentrations of tRNA species by encoding tRNA genes and adapt translation processes to its own codon. In addition to the tRNA genes, RsoM2USA genome was also predicted to encode a tRNA (Ile)-lysidine synthase (ORF84) and glutamyl-tRNA synthase (ORF156) (Table 2), respectively, that may function in tRNA maturation.

Since the genome of RsoM2USA was annotated to encode replication, translation, transcription, 44 tRNA, and tRNA processing enzyme genes, genome replication of RsoM2USA and RsoM2USA-specific gene expression may be less dependent on the host bacterium. Different from some of the jumbo phages (Ceyssens et al., 2014; Yuan and Gao, 2016), however, no ORFs were annotated for RNA polymerases (RNAPs) in the genome of RsoM2USA. It is well known that RNAPs help phages to start immediate early gene expression and produce viral progeny independent of the host transcriptional process (Ceyssens et al., 2014; Yuan and Gao, 2017). The lack of RNAPs in RsoM2USA may explain the long and slow replication cycle of RSoM2USA with a latent period of more than 4.5 h. *Ralstonia* phage RSL1 (Yamada et al., 2010) and a novel benthic phage infecting *Shewanella* (Wang et al., 2019) also have long latent periods of 150 and 200 min, respectively, without their own RNAPs identified. Alternatively, an unknown but essential phage protein may be involved in transcription of the phage. Jumbo phages may also rely on both the host and phage RNAPs as their transcription strategy, such as in the case of Enterobacteria phage N4, where early and middle stage of transcription depends on two phage-encoded RNAPs and the late genes are transcribed using host RNAP (Haynes and Rothman-Denes, 1985; Willis et al., 2002).

#### Structure Proteins

Fifteen ORFs were predicted to be involved in morphogenesis and structure of phage RsoM2USA (Table 2 and Supplementary Table 3). They all shared aa sequence homology with their corresponding ORFs in other phages including Xanthomonas phage XacN1, Agrobacterium phage Atu_ph07, Pectinobacterium phage CBB, and Salicola phage SCTP-2 (Table 2). ORFs 359, 406, 410, and 415 were annotated to encode tail and tail fiber proteins, ORFs 356, 361, 367, 379, and 383 for structure and head morphologies, ORFs 362, 363, 364, and 375 for baseplate wedge and hub proteins, and ORFs 378 and 401 for portal and neck proteins.

**FIGURE 5:**
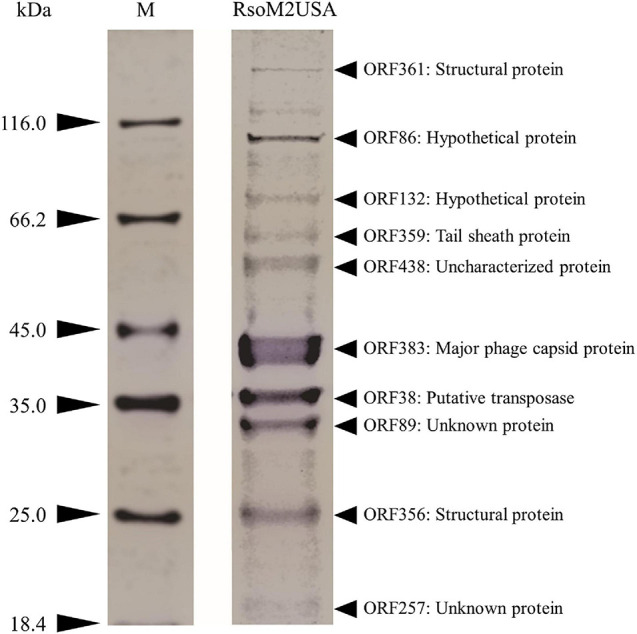
Expression of structural proteins of *Ralstonia* jumbo phage RsoM2USA. Proteins from purified phage virions were separated by SDS-PAGE gel (12%) and stained with Coomassie blue. The functions of the 10 protein bands predicted to correspond to ORFs 38, 86, 89, 132, 257, 356, 359, 361, 383, and 438. M: protein ladder, with its molecular weight in kilodaltons (kDa) indicated on the left.

To confirm the identity of the major structural components of phage RsoM2USA, we performed a proteomic analysis of the purified phage virions by SDS-PAGE gel. At least 10 proteins ranging from 18 to over 116 kDa were separated in the gel (Figure 5). As expected, the most abundant protein observed in the virion proteome is the major capsid protein (ORF383) with a molecular mass of 44.04 kDa (Figure 5). The 9 other bands most likely correspond to structure (ORF361, > 288 kDa; and ORF356, 21.1 kDa), tail sheath (ORF359, 55.7 kDa), putative transposase (ORF38, 37.8 kDa), hypothetical (ORFs 86, 96.44 kDa; ORF132, 76.64 kDa), and unknown or uncharacterized (ORFs 89, 28.57 kDa, 257, 17.57 KDa; and 438, 51.69 kDa) proteins identified based on the method of Addy et al. (2019) by using mass spectrometry and their relative abundance, as well as comparison to the deduced amino acid sequences and molecular masses of proteins predicted from the ORFs in phage RsoM2USA (Figure 5).

### Jumbo Phage RsoM2USA Significantly Reduced the Virulence of Its Susceptible *R. solanacearum* Strain RUN302 in Tomato Plants

**FIGURE 6:**
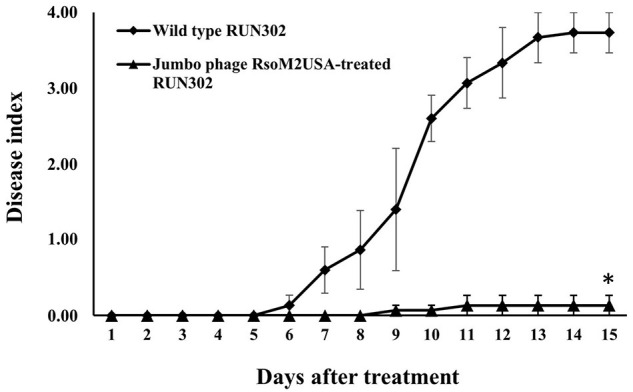
Virulence of *R. solanacearum* strain RUN302 alone (closed diamond) and co-inoculated with jumbo phage RsoM2USA at the MOI of 1 (closed triangle). Disease severity was calculated based on a 0 (healthy) to 4 (75–100% leaves wilted) disease index of each plant. Points shown are means of three separate experiments, each containing 5 plants per treatment. Bars indicate standard errors. Significance of means was measured using the *t*-test (*p* < 0.05) in Microsoft Excel and denoted by an asterisk for statistically significant difference at the indicated timepoint.

To study the effect of the jumbo phage RsoM2USA on the virulence of its susceptible *Rssc* strain RUN302, we compared the virulence of the wild type RUN302 to that of the phage-treated RUN302. The wild type strain RUN302 started to wilt tomato plants 6 days after inoculation with an average DI of 0.4 + 0.2 (*n* = 15), and 14 of the 15 inoculated plants were completely wilted 2 weeks after inoculation (Figure 6). When tomato seedlings were inoculated with the jumbo phage RsoM2USA-treated RUN302 strain, however, only two of the 15 inoculated plants displayed wilt symptom with an average DI of 0.13 + 0.23 even 15 days after inoculation (Figure 6).

Our virulence result is similar to those of *Ralstonia* jumbo phages RSL1 (Fujiwara et al., 2011) and RSF1 (Bhunchoth et al., 2016), as well as non-jumbo phages P4282 (Tanaka et al., 1990), PE204 (Bae et al., 2012), and ϕRSM3 (Addy et al., 2012), showing either total loss of virulence (RSL1, PE204, and ϕRSM3) or reduced virulence (RSF1 and P4284) of their susceptible *Ralstonia* strains in tomato or tobacco (P4282) plants under greenhouse conditions. Recently, three lytic phages were isolated from environmental water in Spain, and the phages either alone or in combination were found effective to control diseases caused by *R. solanacearum* (Álvarez et al., 2019). The effect of the other three *Ralstonia* jumbo phages (RP12, RP31, and RSL2) on virulence, however, remains unknown (Bhunchoth et al., 2016; Matsui et al., 2017). Different from RsoM2USA, *Ralstonia* phage RsoM1USA (Addy et al., 2019) had no significant effect on disease symptoms, although both are myoviruses and isolated from soil in Florida, United States, suggesting that different phages may play different ecological roles in the environment.

*Ralstonia* phages isolated from Japan, Korea and Thailand are only known to be active against *R. pseudosolanacearum* (Álvarez and Biosca, 2017; Addy et al., 2019), so it is hard to assess their biocontrol potential to other *Ralstonia* species. Recently, *Ralstonia* phage RsoP1EGY from Egypt has been found to be specific to only the race 3 biovar 2 strains of *R. solanacearum* (Ahmad et al., 2018), while RsoP1IDN from Indonesia (Addy et al., 2018), RsoM1USA from United States (Addy et al., 2019), and the three lytic phages from Spain (Álvarez et al., 2019) are all active against both *R. solanacearum* and *R. pseudosolanacearum*. The ability of jumbo phage RsoM2USA to significantly reduce the virulence of its bacterial host *R. solanacearum* strain RUN302, the wide host range RsoM2USA displayed, and the specificity of RsoM2USA against *R. solanacearum*, *R. pseudosolanacearum*, and *R. syzygii* make it worthy of future study to determine its potential as biocontrol, either alone or in combination with other compatible phages, against bacterial diseases caused by *Rssc* strains.

## Conclusion

A jumbo *Ralstonia*-infecting phage designated RsoM2USA was isolated from soil in the United States. It belongs to *Myoviridae* with an unusually long latent period of 4.5 h and a burst size of about 32 particles per cell. Its genome contains 343,806 bp with 486 ORFs encoding genes for replication, translation, transcription, 44 tRNAs, and experimentally confirmed structural proteins, as well as many genes whose functions remain to be unraveled. Phylogenetic analyses placed RsoM2USA in the same clade as *Xanthomonas* jumbo phage XacN1, prompting a proposal of a new genus for the two jumbo phages. Phage RsoM2USA displayed a wide host specificity and significantly reduced the virulence of *R. solanacearum* strain RUN302, making it potentially a good candidate for the development of a biocontrol agent against diseases caused by *Rssc* strains.

## Data Availability Statement

The datasets presented in this study can be found in online repositories. The names of the repository/repositories and accession number(s) can be found in the article/Supplementary Material.

## Author Contributions

AA, HA, and QH conceived, designed the experiments, analyzed the data, and wrote the manuscript. AA and HA performed the experiments. QH contributed to the reagents, materials, and analysis tools. All authors contributed to the article and approved the submitted version.

## Conflict of Interest

The authors declare that the research was conducted in the absence of any commercial or financial relationships that could be construed as a potential conflict of interest.

## Publisher’s Note

All claims expressed in this article are solely those of the authors and do not necessarily represent those of their affiliated organizations, or those of the publisher, the editors and the reviewers. Any product that may be evaluated in this article, or claim that may be made by its manufacturer, is not guaranteed or endorsed by the publisher.

## Footnotes

1
http://lowelab.ucsc.edu/tRNAscan-SE/

2
www.megasoftware.net

3
https://talk.ictvonline.org/taxonomy/
