# Supplementary material for: Biological and Molecular Characterization of a Jumbo Bacteriophage Infecting Plant Pathogenic Ralstonia solanacearum Species Complex Strains
Source: Front Microbiol. 2021 Sep 27;12:741600. doi: 10.3389/fmicb.2021.741600 (PMC8504454; doi:10.3389/fmicb.2021.741600)

Supplementary Figure S1. Genome organization of the *Ralstonia* jumbo phage RsoM2USA. On the outer ring, annotated ORFs with predicted functions listed in Table 2 are labeled. On the middle ring, the GC content relative to the mean GC content of the phage is shown. On the inner ring, green represents positive while purple negative GC skew.

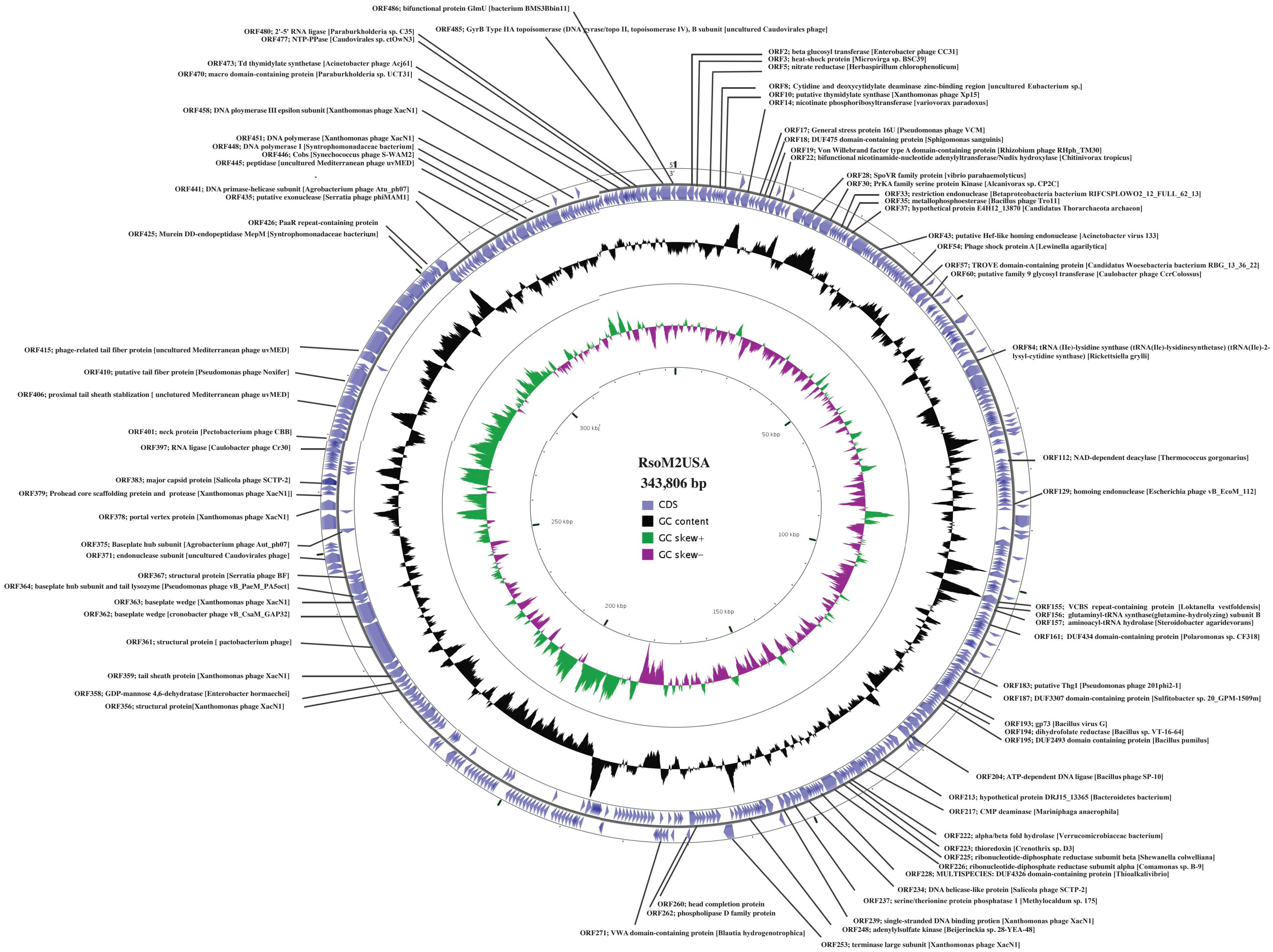

Supplement: Supplementary file 1 [file Data_Sheet_1.zip › Supplementary Figure S1.pdf]
