# Supplementary material for: Biological and Molecular Characterization of a Jumbo Bacteriophage Infecting Plant Pathogenic Ralstonia solanacearum Species Complex Strains
Source: Front Microbiol. 2021 Sep 27;12:741600. doi: 10.3389/fmicb.2021.741600 (PMC8504454; doi:10.3389/fmicb.2021.741600)

Supplementary Fig. S2. Genome comparison between Ralstonia jumbo phage RsoM2USA and 46 representative jumbo phages in the family of *Myoviridae*. The dot plot was generated in Gepard ver 1.40. Other previously published Ralstonia jumbo phages used for the comparison are underlined.

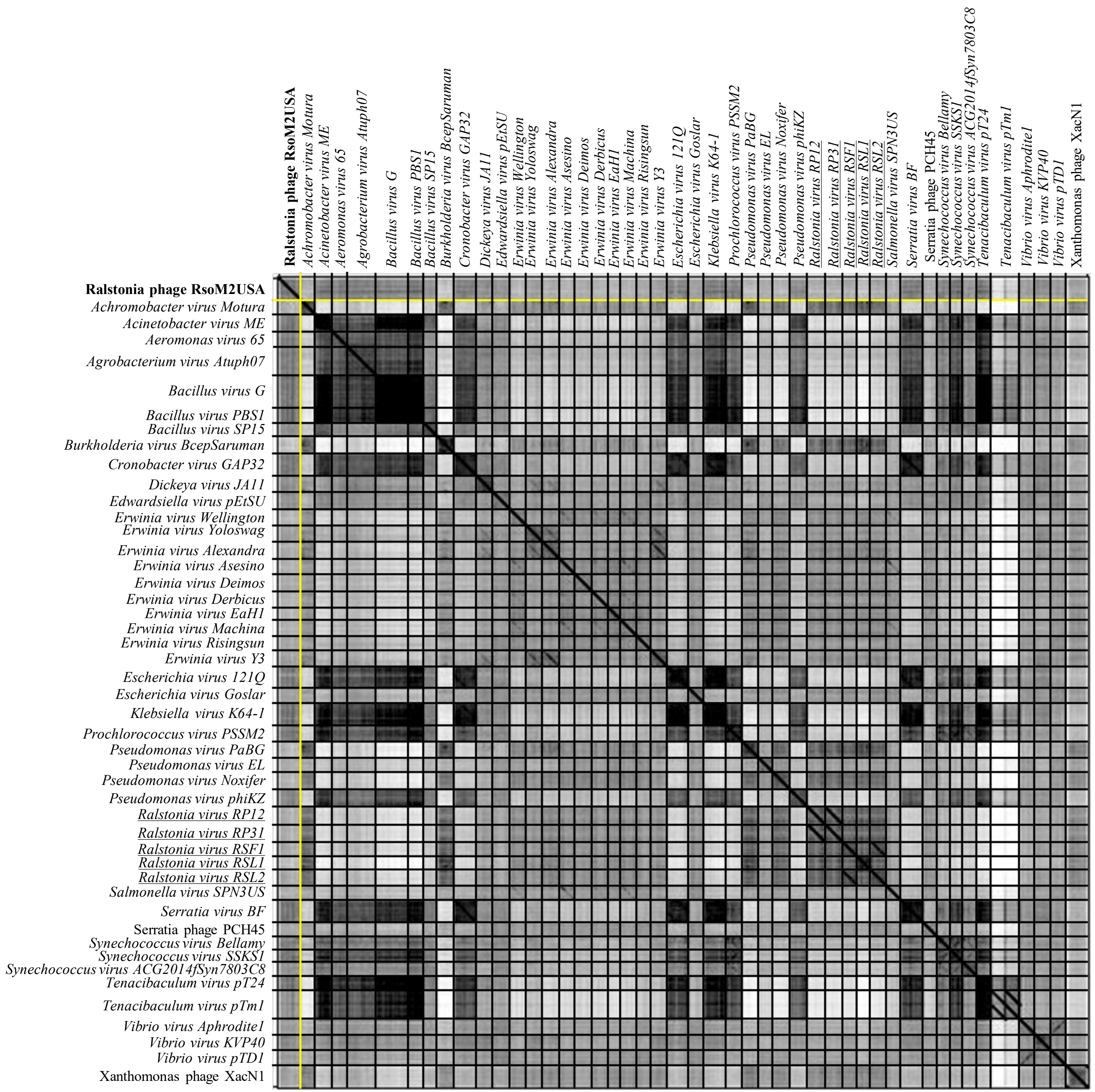

Supplement: Supplementary file 1 [file Data_Sheet_1.zip › Supplementary Figure S2.PDF]
