# Supplementary material for: Biological and Molecular Characterization of a Jumbo Bacteriophage Infecting Plant Pathogenic Ralstonia solanacearum Species Complex Strains
Source: Front Microbiol. 2021 Sep 27;12:741600. doi: 10.3389/fmicb.2021.741600 (PMC8504454; doi:10.3389/fmicb.2021.741600)
Supplement: Supplementary file 1 [file Data_Sheet_1.zip › Supplementary Table S1.PDF]

Supplementary Table S1. List of taxonomy, genome sizes and accession numbers of 47 jumbo phages in the family of *Myoviridae* used in this study for generating dot-plot and calculating Orthologous Average Nucleotide Identity (OrthoANI) values.

Subfamily	Genus	Species	Jumbo phage	Genome Size (bp)	Accession no.
Emmerichvirinae	*Ishigurovirus*	*Aeromonas virus 65*	Aeromonas phage 65	235,229	NC_015251
Gorgonvirinae	*Aphroditevirus*	*Vibrio virus Aphrodite1*	Vibrio phage Aphrodite1	237,722	NC_042100.1
	Tidunavirus	*Vibrio virus pTD1*	Vibrio phage pTD1	239,276	NC_041916.1
Tevenvirinae	*Schizotequatrovirus*	*Vibrio virus KVP40*	Vibrio phage KVP40	244,834	NC_005083.2
UD (Undefined)	*Moturavirus*	*Achromobacter virus Motura*	Achromobacter phage Motura	221,431	NC_049849.1
	Metrivirus	*Acinetobacter virus ME3*	Acinetobacter phage vB_AbaM_ME3	234,900	NC_041884.1
	Polybotosvirus	*Agrobacterium virus Atuph07*	Agrobacterium phage Atuph07	490,380	NC_042013.1
	Donellivirus	*Bacillus virus G*	Bacillus phage G	497,513	NC_023719.1
	Takahashivirus	*Bacillus virus PBS1*	Bacillus phage PBS1	252,197	NC_043027.1
	Thornevirus	*Bacillus virus SP15*	Bacillus phage SP-15	221,908	NC_031245.1
	Sarumanvirus	*Burkholderia virus BcepSaruman*	Burkholderia phage BcepSaruman	263,735	NC_049850.1
	Mimasvirus	*Cronobacter virus GAP32*	Cronobacter phage GAP32	358,663	NC_019401.1
	Salmondvirus	*Dickeya virus JA11*	Dickeya phage vB_DsoM_JA11	255,356	NC_048077.1
	Petsuvirus	*Edwardsiella virus pEtSU*	Edwardsiella phage pEt-SU	276,734	NC_048182.1
	Alexandravirus	*Erwinia virus Alexandra*	Erwinia phage vB_EamM_Alexandra	266,532	NC_047995.1
	Erskinevirus	*Erwinia virus Asesino*	Erwinia phage vB_EamM_Asesino	246,290	NC_031107.2
	Derbicusvirus	*Erwinia virus Derbicus*	Erwinia phage Derbicus	223,950	NC_048173.1
	Agricanvirus	*Erwinia virus Deimos*	Erwinia phage vB_EamM_Deimos-Minion	273,501	NC_041972.1
	Iapetusvirus	*Erwinia virus EaH1*	Erwinia phage PhiEaH1	218,339	NC_023610.1
	Machinavirus	*Erwinia virus Machina*	Erwinia phage vB_EamM_Machina	241,654	NC_042056.1
	Risingsunvirus	*Erwinia virus Risingsun*	Erwinia phage vB_EamM_Risingsun	235,108	NC_042018.1
	Wellingtonvirus	*Erwinia virus Wellington*	Erwinia phage vB_EamM_Wellington	244,950	NC_048016.1
	Sasquatchvirus	*Erwinia virus Y3*	Erwinia phage vB_EamM_Y3	261,365	NC_047880.1
	Yoloswagvirus	*Erwinia virus Yoloswag*	Erwinia phage vB_EamM_Yoloswag	259,700	NC_047815.1
	Asteriusvirus	*Escherichia virus 121Q*	Escherichia phage 121Q	348,532	NC_025447.1
	Goslarvirus	*Escherichia virus Goslar*	Escherichia phage vB_EcoM_Goslar	237,307	NC_048170.1
	Alcyoneusvirus	*Klebsiella virus K64-1*	Klebsiella phage K64-1	346,602	NC_027399.1

Subfamily	Genus	Species	Jumbo phage	Genome Size (bp)	Accession no.
	Salacisavirus	*Prochlorococcus virus PSSM2*	Prochlorococcus phage P-SSM2	252,401	NC_006883.2
	Elvirus	*Pseudomonas virus EL*	Pseudomonas phage EL	211,215	NC_007623.1
	Noxifervirus	*Pseudomonas virus Noxifer*	Pseudomonas phage Noxifer	278,136	NC_041994.1
	Baikalvirus	*Pseudomonas virus PaBG*	Pseudomonas phage PaBG	258,139	NC_022096.1
	Phikzvirus	*Pseudomonas virus phiKZ*	Pseudomonas phage phiKZ	280,334	NC_004629.1
	Ripduovirus	*Ralstonia virus RP12*	Ralstonia phage RP12	279,845	NC_041911.1
		Ralstonia virus RP31	Ralstonia phage RP31	276,958	AP017925.1
	Chiangmaivirus	*Ralstonia virus RSF1*	Ralstonia phage RSF1	222,888	NC_028899.1
		Ralstonia virus RSL2	Ralstonia phage RSL2	223,932	NC_028950.1
	Mieseafarmvirus	*Ralstonia virus RSL1*	Ralstonia phage RSL1	231,255	NC_010811.2
	Seoulvirus	*Salmonella virus SPN3US*	Salmonella phage SPN3US	240,413	NC_027402.1
	Eneladusvirus	*Serratia virus BF*	Serratia phage BF	357,154	NC_041917.1
	Atlauavirus	*Synechococcus virus AC2014fSyn7803C8*	Synechococcus phage ACG-2014f	223,270	NC_047713.1
	Bellamyvirus	*Synechococcus virus Bellamy*	Synechococcus phage Bellamy	204,930	NC_047838.1
	Llyrvirus	*Synechococcus virus SSKS1*	Synechococcus phage S-SKS1	208,007	NC_020851.1
	Kungbxnavirus	*Tenacibaculum virus pT24*	Tenacibaculum phage pT24	234,670	NC_049383.1
	Shirahamavirus	*Tenacibaculum virus pTm1*	Tenacibaculum phage PTm1	224,680	NC_049340.1
	UD	UD	Xanthomonas phage XacN1	384,670	AP018399.1
			Serratia phage PCH45	212,807	MN334766
			Ralstonia phage RsoM2USA	343,806	MG752970
