# Supplementary material for: Biological and Molecular Characterization of a Jumbo Bacteriophage Infecting Plant Pathogenic Ralstonia solanacearum Species Complex Strains
Source: Front Microbiol. 2021 Sep 27;12:741600. doi: 10.3389/fmicb.2021.741600 (PMC8504454; doi:10.3389/fmicb.2021.741600)
Supplement: Supplementary file 1 [file Data_Sheet_1.zip › Supplementary Table S2..PDF]

Supplementary Table S2. List of jumbo phages in the family of *Myoviridae* and their genome and protein (ORF) accession numbers used for phylogenetic analyses in this study

Subfamily	Genus	Species	Jumbo phage	Genome Size (bp)	Accession No			
					Phage Genome	Major phage capsid protein	Terminase large subunit protein	Portal vertex protein
Emmerichvirinae	*Ishigurovirus*	*Aeromonas virus 65*	Aeromonas phage 65	235,229	NC_015251	YP_004300919.1	YP_004300928.1	YP_004300925.1
Tevenvirinae	*Schizotequatrovirus*	*Vibrio virus KVP40*	Vibrio phage KVP40	244,834	NC_005083.2	NP_899609.1	NP_899601.1	NP_899604.1
		Vibrio virus nt1	Vibrio phage nt-1	247,511	NC_021529.2	YP_008125183.1	YP_008125177.1	YP_008125180.1
		Vibrio virus ValKK3	Vibrio phage ValKK3	248,088	NC_028829.1	YP_009201384.1	YP_009201392.1	YP_009201389.1
UD	*Metrivirus*	*Acinetobacter virus ME3*	Acinetobacter phage vB_AbaM_ME3	234,900	NC_041884.1	AND75183.1	AND75174.1	AND75180.1
	Mimasvirus	*Cronobacter virus GAP32*	Cronobacter phage GAP33	358,663	NC_019401.1	AFC21700.1	AFC21719.1	AFC21695.1
		Pectinobacterium virus CBB	Pectinobacterium phage CBB	378,379	NC_041878.1	AMM43821.1	AMM43839.1	MM43817.1
	Petsuvirus	*Edwardsiella virus pEtSU*	Edwardsiella phage pEt-SU	276,734	NC_048182.1	YP_009821962.1	YP_009821923.1	NF
	Asteriusvirus	*Escherichia virus 121Q*	Escherichia phage 121Q	348,532	NC_025447.1	YP_009102190.1	YP_009101598.1	YP_009102185.1
		Escherichia virus PBECO4	Escherichia phage PBECO4	348,113	NC_027364.1	YP_009150832.1	YP_009150814.1	YP_009150837.1
	Alcyoneusvirus	*Klebsiella virus K64-1*	Klebsiella phage K64-1	346,602	NC_027399.1	NF	YP_009153167.1	YP_009153162.1
		Klebsiella virus RaK2	Klebsiella phage vB_KleM_RaK2	345,809	NC_019526.1	YP_007007249.1	YP_007007266.1	YP_007007244.1
	Salacisavirus	*Prochlorococcus virus PSSM2*	Prochlorococcus phage P-SSM2	252,401	NC_006883.2	ACY76014.1	ACY76007.1	NF
	Ripduovirus	*Ralstonia virus RP12*	Ralstonia phage RP12	279,845	NC_041911.1	YP_009598787.1	YP_009598717.1	NF
		Ralstonia virus RP31	Ralstonia phage RP31	276,958	AP017925.1	BAW19353.1	BAW19286.1	NF
	Chiangmaivirus	*Ralstonia virus RSF1*	Ralstonia phage RSF1	222,888	NC_028899.1	YP_009207928.1	YP_009207830.1	NF
		Ralstonia virus RSL2	Ralstonia phage RSL2	223,932	NC_028950.1	YP_009212966.1	YP_009212872.1	NF
	Mieseafarmvirus	*Ralstonia virus RSL1*	Ralstonia phage RSL1	231,255	NC_010811.2	YP_001950011.1	YP_001950095.1	YP_001950090.1
	Eneladusvirus	*Serratia virus BF*	Serratia phage BF	357,154	NC_041917.1	YP_009599751.1	YP_009599769.1	YP_009599746.1
		Yersinia virus Yen9-04	Yersinia phage fHe-Yen9-04	354,378	NC_042116.1	SOK58527.1	SOK58545.1	SOK58522.1
	Bellamyvirus	*Synechococcus virus Bellamy*	Synechococcus phage Bellamy	204,930	NC_047838.1	YP_009791290.1	YP_009791281.1	YP_009791285.1
	Llyrvirus	*Synechococcus virus SSKS1*	Synechococcus phage S-SKS1	208,007	NC_020851.1	YP_007674500.1	YP_007674510.1	YP_007674507.1
	Shirahamavirus	*Tenacibaculum virus pTm1*	Tenacibaculum phage PTm1	224,680	NC_049340.1	YP_009873985.1	YP_009873697.1	YP_009873982.1
	UD	UD	Xanthomonas phage XacN1	384,670	AP018399.1	BBA65422.1	BBA65403.1	BBA65449.1
			Ralstonia phage RsoM2USA	343,806	MG752970	AVH85311	AVH85181	AVH85306

UD: Undefined. NF: Annotation for the protein in the jumbo phage genome has not been found.
