# Supplementary material for: Biological and Molecular Characterization of a Jumbo Bacteriophage Infecting Plant Pathogenic Ralstonia solanacearum Species Complex Strains
Source: Front Microbiol. 2021 Sep 27;12:741600. doi: 10.3389/fmicb.2021.741600 (PMC8504454; doi:10.3389/fmicb.2021.741600)
Supplement: Supplementary file 1 [file Data_Sheet_1.zip › Supplementary Table S3.PDF]

	A	B	C	D	E	F	G	H	I
1	**Supplementary Table S3. Genome annotation of Ralstonia jumbo phage RsoM2USA.**								
2	**ORF**	**Strand**	**Position 5'-3'**		**Length of Protein (aa)**	**Amino acid sequence identity/similarity to homologs (Query cover %; no. of amino acid identical; % identity)**	**E-value**	**Accession no.**	**Identity%**
3			start	end					
4	ORF1	-1	29	1888	620	Yvck Family protein [Enterocloster citroniae]	3.00E-82	WP_083423907.1	36
5	ORF2	-2	1890	3020	377	beta glucosyl transferase [Enterobacter phage CC31]	5.00E-38	YP_004009897.1	30
6	ORF3	-3	3079	3714	212	heat-shock protein [Microvirga sp. BSC39]	5.00E-22	WP_036354424.1	44
7	ORF4	-2	3810	5141	444	tetratricopeptide repeat protein [Rhodospirillaceae bacterium]	5.00E-40	MSO88909.1	30
8	ORF5	-1	5117	5956	280	nitrate reductase [Herbaspirillum chlorophenolicum]	6.00E-69	WP_050467745.1	43
9	ORF6	-3	5956	6585	210	gp315 [Bacillus virus G]	5.00E-04	YP_009015618.1	28
10	ORF7	-2	6582	6890	103	GIY-YIG nuclease family protein [Wenzhouxiangella sp. C33]	7.00E-09	WP_164211623.1	32
11	ORF8	-2	6927	7346	140	cytidine and deoxycytidylate deaminase zinc-binding region [uncultured Eubacterium sp.]	1.00E-26	SCI18574.1	39
12	ORF9	-2	7383	7607	75	hypothetical protein [Azospirillum oryzae]	6.00E-21	WP_085085423.1	57
13	ORF10	-	7607	8443	279	putative thymidylate synthase [Xanthomonas phage Xp15]	2.00E-50	YP_239304.1	39
14	ORF11	-1	8534	9907	458	hypothetical protein FLAPJACK_212 [Bacillus phage Flapjack]	4.00E-19	ARQ95137.1	33
15	ORF12	-1	9926	10729	268	hypothetical protein PBI_SCTP2_386 [Salicola phage SCTP-2]	6.00E-64	ASV44401.1	45
16	ORF13	+	10367	11068	234	No significant found			
17	ORF14	-3	10780	12177	466	nicotinate phosphoribosyltransferase [Variovorax paradoxus]	0	WP_081267491.1	61
18						nicotinate phosphoribosyltransferase [Ralstonia solanacearum]	0	WP_094395128.1	58
19	ORF15	-	12298	12702	135	No significant found			
20	ORF16	-3	12709	13182	158	NUDIX hydrolase [Candidatus Thorarchaeota archaeon]	2.00E-34	TFG98295.1	48
21	ORF17	-2	13266	13880	205	general stress protein 16U [Pseudomonas phage VCM]	2.00E-45	YP_009222754.1	44
22	ORF18	-3	13867	14931	355	DUF475 domain-containing protein [Sphingomonas sanguinis]	7.00E-98	WP_058733334.1	46

	A	B	C	D	E	F	G	H	I
23	ORF19	-1	14888	15565	226	Von Willebrand factor type A domain-containing protein [Rhizobium phage RHph_TM30]	6.00E-86	QIG71336.1	56
24	ORF20	-1	15566	16699	378	toxic anion resistance protein [Rhizobium phage RHph_TM30]	1.00E-88	QIG71335.1	42
25						Toxic anion resistance protein (TelA) [Pseudomonas phage VCM]	3.00E-73	YP_009222751.1	34
26	ORF21	+	16449	16742	98	No significant found			
27	ORF22	-2	16776	17813	346	bifunctional nicotinamide-nucleotide adenylyltransferase/Nudix hydroxylase [Chitinivorax tropicus]	1.00E-96	WP_184035971.1	45
28						von Willebrand factor type A domain protein [Pseudomonas phage VCM]	3.00E-50	YP_009222752.1	46
29	ORF23	-2	17892	18233	114	No significant similarity found			
30	ORF24	-1	18452	19123	224	No significant similarity found			
31	ORF25	+	18560	19144	195	No significant found			
32	ORF26	-2	19902	21473	524	hypothetical protein FDI40_gp196 [Agrobacterium phage Atu_ph07]	2.00E-23	YP_009611861.1	36
33	ORF27	-2	21558	21857	100	hypothetical protein CcrColossus_gp171 [Caulobacter phage CcrColossus]	1.00E-04	AFU88041.1	34
34	ORF28	-1	21854	23371	506	SpoVR family protein [Vibrio parahaemolyticus].	0	WP_025610357	56
35	ORF29	-3	23368	24654	429	YeaH/YhbH family protein [Gammaproteobacteria bacterium]	6.00E-111	MBN9289226.1	44
36	ORF30	-3	24727	26670	648	PrkA family serine protein kinase [Alcanivorax sp. CP2C]	0	WP_067606528.1	61
37	ORF31	-1	26684	27319	212	AAA family ATPase [Planctomycetes bacterium]	8.00E-45	MBI2900133.1	49
38	ORF32	-	27324	27527	68	No significant similarity found.			
39	ORF33	-1	27527	28144	206	restriction endonuclease [Betaproteobacteria bacterium RIFCSPLOWO2_12_FULL_62_13]	5.00E-55	OGA37234.1	61
40	ORF34	-2	28212	28478	89	No significant similarity found			
41	ORF35	-1	28475	29101	209	metallophosphoesterase [Bacillus phage Troll]	7.00E-32	YP_008430899.1	39
42	ORF36	-1	29123	29545	141	hypothetical protein [Ralstonia phage RSP15]	3.00E-09	YP_009276981.1	27
43	ORF37	-2	29535	29810	92	hypothetical protein E4H12_13870 [Candidatus Thorarchaeota archaeon]	1.00E-13	TFG95161.1	42
44	ORF38	-1	30020	31024	335	transposase [Sphingobium phage S21]-430aa	9.00E-52	ARK07475.1	40

	A	B	C	D	E	F	G	H	I
45						protector from prophage-induced early lysis [Escherichia phage vB_EcoM_112]/312 aa	2.00E-46	YP_009030884.1	40
46	ORF39	-2	31017	33407	797	hypothetical protein UFOVP29_118 [uncultured Caudovirales phage]	5.00E-59	CAB4122959.1	34
47	ORF40	-1	33404	33739	112	hypothetical protein [Brevibacillus brevis]	1.00E-04	WP_017248731.1	30
48	ORF41	-1	33851	34090	80	No significant similarity found			
49	ORF42	-3	34162	34623	154	hypothetical protein AAY80_119 [Stenotrophomonas phage vB_SmaS-DLP_6]	3.00E-25	AMQ66021.1	47
50	ORF43	-1	34664	35284	207	putative Hef-like homing endonuclease [Acinetobacter virus 133]	1.00E-06	YP_004300760.1	47
51	ORF44	-2	35281	36216	312	hypothetical protein [Bdellovibrionales bacterium]	1.00E-12	HAG92078.1	27
52	ORF45	-2	36213	37292	360	MULTISPECIES: hypothetical protein [Achromobacter]	7.00E-06	WP_026382941.1	45
53	ORF46	-3	37270	37833	188	No significant similarity found			
54	ORF47	-1	37775	38437	221	hypothetical protein [Bacteroidia bacterium]	6.00E-10	MBT8232621.1	30
55	ORF48	-2	38421	38789	123	No significant similarity found			
56	ORF49	-1	38786	39535	250	GNAT family N-acetyltransferase [Tibeticola sediminis]	1.00E-04	WP_124222602.1	27
57	ORF50	-3	39532	39858	109	No significant similarity found			
58	ORF51	-2	40035	40202	56	hypothetical protein vB_PsyM_KIL2_0016 [Pseudomonas phage vB_PsyM_KIL2]	1.00E-04	AMR57427.1	45
59	ORF52	-1	40202	41104	301	hypothetical protein B0E49_09960 [Polaromonas sp. C04]	1.00E-73	OOG54330.1	46
60						C4-dicarboxylate ABC transporter substrate-binding protein [Rhodovulum sulfidophilum]	1.00E-46	OLS50414.1	36
61	ORF53	-1	41135	41503	123	No significant similarity found			
62	ORF54	-2	41514	42440	309	hypothetical protein [Paraburkholderia phenazinium]	3.00E-65	WP_074295960.1	37
63						Phage shock protein A [Lewinella agarilytica]	1.00E-22	SEQ17255.1	29
64	ORF55	-2	42444	43079	212	No significant similarity found			
65	ORF56	-1	43115	43519	135	gp116 [Bacillus virus G]	2.00E-15	YP_009015419.1	34
66	ORF57	-3	43612	45078	489	TROVE domain-containing protein [Candidatus Woesebacteria bacterium RBG_13_36_22]	5.00E-143	OGM09089.1	50
67						putative TROVE-like domain protein [Caulobacter phage CcrColossus]	4.00E-106	YP_006988373.1	43
68	ORF58	+	45234	45437	68	No significant similarity found			
69	ORF59	-1	45395	46471	359	No significant similarity found			

	A	B	C	D	E	F	G	H	I
70	ORF60	-3	46489	47295	269	hypothetical protein [Pseudomonas luteola]	2.00E-33	WP_019364306.1	35
71						putative family 9 glycosyl transferase [Caulobacter phage CcrColossus]	5.00E-17	YP_006988365.1	37
72	ORF61	+	47317	48027	237	hypothetical protein [Pandoraea morbifera]	1.00E-06	WP_150565314.1	34
73	ORF62	-3	48004	48465	154	hypothetical protein [Methylocystis bryophila]	4.00E-44	WP_085773879.1	65
74						hypothetical protein CcrColossus_gp169 [Caulobacter phage CcrColossus]	1.00E-39	YP_006988403.1	62
75	ORF63	-1	48467	48697	77	No significant similarity found			
76	ORF64	-2	48657	48971	105	No significant similarity found			
77	ORF65	2	49031	49522	164	No significant similarity found			
78	ORF66	-1	49496	49690	65	No significant similarity found			
79	ORF67	-	50399	50623	75	No significant similarity found			
80	ORF68	-1	50645	51307	221	No significant similarity found			
81	ORF69	-3	51358	51795	146	No significant similarity found			
82	ORF70	-2	51858	52514	219	No significant similarity found			
83	ORF71	-2	52567	53655	363	hypothetical protein [Acetobacter malorum]	5.00E-13	WP_061505186.1	27
84	ORF72	+	52651	53682	344	No significant similarity found			
85	ORF73	-	53688	54110	141	No significant similarity found			
86	ORF74	-1	53774	54235	154	No significant similarity found			
87	ORF75	-3	54235	54789	185	hypothetical protein [Diaphorobacter polyhydroxybutyrativorans]	3.00E-53	WP_088887310.1	56
88						putative 5'3'-deoxyribonucleotidase [Cronobacter phage vB_CsaM_GAP32]	2.00E-29	YP_006987476.1	46
89	ORF76	-1	54935	55132	66	No significant similarity found			
90	ORF77	-2	55239	55469	77	hypothetical protein COB55_03030 [Candidatus Wolfebacteria bacterium]	5.00E-36	PCI28923.1	75
91	ORF78	-1	55466	55714	83	No significant similarity found			
92	ORF79	+	55743	55958	72	No significant similarity found			
93	ORF80	-3	56032	57231	400	glycosyltransferase [Myoviridae sp.]	3.00E-109	AXH72901.1	48
94	ORF81	-3	57328	57867	180	No significant similarity found			
95	ORF82	-2	57864	58508	215	No significant similarity found			
96	ORF83	+	59076	59294	73	No significant similarity found			

	A	B	C	D	E	F	G	H	I
97	ORF84	-3	59431	60477	349	tRNA(Ile)-lysidine synthase (tRNA(Ile)-lysidinesynthetase) (tRNA(Ile)-2-lysyl-cytidine synthase) [Rickettsiella grylli]	2.00E-32	EDP46981.1	52
98	ORF85	-1	60506	62338	611	hypothetical protein [Syntrophomonadaceae bacterium]	5.00E-150	MBT9137776.1	41
99	ORF86	-3	62335	64857	841	hypothetical protein [Syntrophomonadaceae bacterium]	0	MBT9137777.1	41
100	ORF87	-2	64905	65090	62	No significant similarity found			
101	ORF88	-	65163	66029	289	No significant similarity found			
102	ORF89	-2	66030	66791	254	No significant similarity found			
103	ORF90	-1	66788	67654	289	No significant similarity found			
104	ORF91	-	67720	68259	180	hypothetical protein [Pseudomonas amygdali]	2.00E-60	WP_005742872.1	51
105	ORF92	-3	68287	68847	187	No significant similarity found			
106	ORF93	-3	69292	69507	72	No significant similarity found			
107	ORF94	-1	69491	69964	158	No significant similarity found			
108	ORF95	-2	69894	70319	142	ATP-binding protein [Vibrio hyugaensis]	1.00E-34	WP_045464763.1	46
109						hypothetical protein FV3_00074 [Escherichia phage FV3]	2.00E-32	YP_007006245.1	49
110	ORF96	-	70285	70494	70	No significant similarity found			
111	ORF97	-2	70491	70844	118	No significant similarity found			
112	ORF98	+	70594	70980	129	No significant similarity found			
113	ORF99	-2	70875	71144	90	No significant similarity found			
114	ORF100	-3	71155	71586	144	hypothetical protein EO766_11665 [Hydrotalea sp. AMD]	8.00E-21	RWZ87186.1	41
115	ORF101	2	71606	71788	61	No significant similarity found			
116	ORF102	-1	71858	72562	235	hypothetical protein [Caballeronia calidae]	3.00E-27	WP_074173624.1	32
117						hypothetical protein AAY80_169 [Stenotrophomonas phage vB_SmaS-DLP_6]	3.00E-13	AMQ65954.1	28
118	ORF103	+	72137	72619	161	No significant similarity found			
119	ORF104	-3	72559	73731	391	hypothetical protein PBI_SCTP2_41 [Salicola phage SCTP-2]	0.00E+00	ASV44056.1	29
120	ORF105	+	72913	73776	288	No significant similarity found			
121	ORF106	+	73730	74191	154	No significant similarity found			
122	ORF107	-3	74272	74595	108	hypothetical protein [Burkholderia ubonensis]	6.00E-05	WP_059928226.1	34
123	ORF108	-2	74667	75038	124	hypothetical protein HOV08_gp054 [Vibrio phage VspSw_1]	6.00E-09	YP_009819571.1	39
124	ORF109	-3	76015	76257	81	No significant similarity found			
125	ORF110	-3	76387	76596	70	No significant similarity found			

	A	B	C	D	E	F	G	H	I
126	ORF111	1	77128	77523	132	No significant similarity found			
127	ORF112	-1	77678	78244	189	NAD-dependent deacylase [Thermococcus gorgonarius]	1.00E-43	WP_088885183.1	42
128	ORF113	-2	78378	78704	109	No significant similarity found			
129	ORF114	-2	78813	79163	117	No significant similarity found			
130	ORF115	-1	79163	79339	77	hypothetical protein [Paraburkholderia sp. Ac-20336]	6.00E-07	WP_205989950.1	47
131	ORF116	-2	79362	80018	219	hypothetical protein [Pectobacterium carotovorum]	9.00E-09	WP_010681489.1	33
132	ORF117	-2	80283	80504	74	No significant similarity found			
133	ORF118	-3	80641	81651	337	hypothetical protein [Ralstonia phage RSP15]	3.00E-91	YP_009277121.1	49
134	ORF119	-	81572	81811	80	hypothetical protein [Pseudomonas phage phiPsa374]	9.00E-16	YP_009009442.1	49
135	ORF120	-3	81811	82011	67	No significant similarity found			
136	ORF121	-3	82084	82599	172	No significant similarity found			
137	ORF122	-1	82763	83008	82	No significant similarity found			
138	ORF123	-2	82992	83264	91	No significant similarity found			
139	ORF124	-1	83624	83854	77	hypothetical protein BF_0461 [Serratia phage BF]	3.00E-18	AQW88986.1	51
140	ORF125	+	83657	83914	86	No significant similarity found			
141	ORF126	-3	84043	84348	102	No significant similarity found.			
142	ORF127	-2	84726	84947	74	hypothetical protein AWU57_360 [Marinobacter sp. T13-3]	4.00E-07	KXS55261.1	38
143	ORF128	-	84944	85210	89	hypothetical protein [archaeon]	9.00E-43	NCP98037.1	77
144	ORF129	-3	85243	85968	242	homing endonuclease [Escherichia phage vB_EcoM_112]	1.00E-23	YP_009030743.1	37
145	ORF130	1	85942	86199	86	No significant similarity found			
146	ORF131	-3	86260	86601	114	hypothetical protein [Bacteroidetes bacterium]	3.00E-32	MBA3680352.1	59
147	ORF132	3	87582	89606	675	hypothetical protein Lu11_0207 [Pseudomonas phage Lu11]	1.00E-48	YP_006382743.1	46
148	ORF133	-1	89639	90049	137	No significant similarity found			
149	ORF134	+	90121	90780	220	hypothetical protein [Euryarchaeota archaeon]	5.00E-21	MBR20447.1	31
150	ORF135	3	90777	91616	280	MULTISPECIES: hypothetical protein [Achromobacter]	2.00E-21	WP_026382940.1	51
151	ORF136	-1	91808	92938	377	hypothetical protein [Verrucomicrobiales bacterium]	4.00E-35	MAB60879.1	37
152	ORF137	-1	92939	93331	131	No significant similarity found			
153	ORF138	-1	93398	93892	165	hypothetical protein [Streptomyces phage BRock]	8.00E-06	APC46355.1	44
154	ORF139	-3	93895	94239	115	hypothetical protein SmphiM12_476 [Sinorhizobium phage phiM12]	2.00E-40	YP_009143283.1	58
155	ORF140	-2	94236	94661	142	No significant similarity found			

	A	B	C	D	E	F	G	H	I
156	ORF141	1	94708	94926	73	No significant similarity found			
157	ORF142	-1	95041	95508	156	No significant similarity found			
158	ORF143	-2	95658	95981	108	No significant similarity found			
159	ORF144	-3	95932	96270	113	No significant similarity found			
160	ORF145	-1	96407	96655	83	No significant similarity found			
161	ORF146	-3	96718	97359	214	hypothetical protein CTY12_06275 [Methylotenera sp.]	2.00E-57	PPD52544.1	46
162	ORF147	-2	97416	98507	364	unnamed protein product [Ralstonia phage phiRSL1]	7.00E-76	YP_001949946.1	74
163	ORF148	1	98485	98781	99	No significant similarity found			
164	ORF149	-2	99087	99722	212	hypothetical protein BCP8-2_026 [Bacillus phage BCP8-2]	4.00E-41	YP_009149587.1	44
165	ORF150	-1	99794	100162	123	hypothetical protein H1O16_gp133 [Burkholderia phage BcepSaruman]	1.00E-18	YP_009904066.1	38
166	ORF151	-2	100131	100337	69	No significant similarity found			
167	ORF152	-3	100378	100797	140	No significant similarity found			
168	ORF153	-2	100800	101297	166	No significant similarity found			
169	ORF154	+	101452	101655	68	No significant similarity found			
170	ORF155	-1	101717	103162	482	VCBS repeat-containing protein [Loktanella vestfoldensis]	7.00E-28	WP_087211375.1	30
171	ORF156	-	103221	103724	168	glutaminyl-tRNA synthase (glutamine-hydrolyzing) subunit B [Candidatus Saccharibacteria bacterium 49-20]	5.00E-08	OJU87614.1	38
172	ORF157	-1	103724	104122	133	aminoacyl-tRNA hydrolase [Steroidobacter agaridevorans]	7.00E-53	WP_202623994.1	65
173	ORF158	-2	104124	104321	66	hypothetical protein E6R13_05235 [Spirochaetes bacterium]	2.00E-08	TXG82274.1	53
174	ORF159	-2	104988	105287	100	No significant similarity found			
175	ORF160	-1	105446	105970	175	hypothetical protein BO221_13845 [Archangium sp. Cb G35]	3.00E-58	OJT24257.1	52
176	ORF161	-2	105942	106757	272	DUF4343 domain-containing protein [Polaromonas sp. CF318]	2.00E-68	WP_007869793.1	42
177	ORF162	1	106771	106965	65	No significant similarity found			
178	ORF163	-3	106939	107316	126	No significant similarity found			
179	ORF164	-2	107313	107618	102	hypothetical protein P26059A_0072 [Curvibacter phage P26059A]	2.00E-05	ASJ79224.1	33
180	ORF165	-3	107578	107889	104	hypothetical protein CcrColossus_gp381 [Caulobacter phage CcrColossus]	5.00E-24	YP_006988615.1	55
181	ORF166	3	107994	108449	152	No significant similarity found			
182	ORF167	-2	107973	108200	76	hypothetical protein [Ralstonia phage RP13]	3.00E-35	BCG50227.1	44

	A	B	C	D	E	F	G	H	I
183	ORF168	-1	108449	108646	66	hypothetical protein UFOVP703_67 [uncultured Caudovirales phage]	3.00E-14	CAB4159176.1	51
184	ORF169	-1	108719	109297	193	hypothetical protein [bacterium]	7.00E-22	NDG31400.1	58
185	ORF170	-2	109353	109589	79	No significant similarity found			
186	ORF171	+	109612	109902	97	hypothetical protein UV79_C0005G0011 [candidate division TM6 bacterium GW2011_GWF2_43_17]	8.00E-04	KKT01765.1	53
187	ORF172	-2	110367	110603	79	hypothetical protein [Noviherbaspirillum massiliense]	9.00E-26	WP_019139920.1	58
188	ORF173	-1	110603	110875	91	hypothetical protein AAY80_048 [Stenotrophomonas phage vB_SmaS-DLP_6]	2.00E-16	AMQ66098.1	46
189	ORF174	-1	110987	111163	59	No significant similarity found			
190	ORF175	-1	111164	111391	76	hypothetical protein [Ralstonia phage RP13]	1.00E-16	BCG50278.1	56
191	ORF176	1	111742	112104	121	No significant similarity found			
192	ORF177	-2	112254	112676	141	hypothetical protein [Sphingobacteriales bacterium]	1.00E-06	MBI3137063.1	29
193	ORF178	-1	112739	113227	163	hypothetical protein DSY47_02760 [Hydrogenothermus sp.]	4.00E-16	RUM49913.1	36
194	ORF179	-3	113224	114036	271	hypothetical protein A3C13_01140 [Candidatus Lloydbacteria bacterium RIFCSPHIGHO2_02_FULL_50_11]	2.00E-36	OGZ08065.1	33
195	ORF180	-1	114125	114430	102	hypothetical protein [Candidatus Roizmanbacteria bacterium]	3.00E-08	HGS99563.1	35
196	ORF181	-2	114438	114695	86	No significant similarity found			
197	ORF182	+	114758	115183	142	No significant similarity found			
198	ORF183	-3	114961	115776	272	putative Thg1 [Pseudomonas phage 201phi2-1]	5.00E-67	YP_001957040.1	44
199	ORF184	-2	115776	116045	90	MULTISPECIES: hypothetical protein [Enterococcus]	2.00E-08	WP_113849445.1	37
200	ORF185	-2	116160	116876	239	No significant similarity found			
201	ORF186	-1	116918	117286	123	No significant similarity found			
202	ORF187	-3	117319	117720	134	DUF3307 domain-containing protein [Sulfitobacter sp. 20_GPM-1509m]	4.00E-19	WP_028956004.1	36
203	ORF188	-2	117717	118202	162	hypothetical protein [Actinobacteria bacterium]	6.00E-47	NCZ69240.1	50
204	ORF189	-1	118199	118465	89	DUF4884 domain-containing protein [Petrimonas mucosa]	9.00E-08	WP_083373188.1	40
205	ORF190	-3	118462	118803	114	No significant similarity found			
206	ORF191	-2	118806	119102	99	No significant similarity found			
207	ORF192	-1	119099	119443	115	No significant similarity found			
208	ORF193	-1	119483	121156	558	gp73 [Bacillus virus G]	3.00E-109	YP_009015384.1	38
209	ORF194	-3	121156	121710	185	dihydrofolate reductase [Bacillus sp. VT-16-64]	1.00E-30	WP_077113372.1	38
210	ORF195	-1	121703	122092	130	DUF2493 domain-containing protein [Bacillus pumilus]	3.00E-25	WP_074041829.1	52

	A	B	C	D	E	F	G	H	I
211						hypothetical protein Phi4:1_gp036 [Cellulophaga phage phi4:1]	3.00E-22	YP_008240624.1	51
212	ORF196	-3	122143	122658	172	hypothetical protein [Lake Baikal phage Baikal-20-5m-C28]	1.00E-37	ATV46347.1	46
213	ORF197	-2	122727	123104	126	No significant similarity found			
214	ORF198	-1	123218	123463	82	No significant similarity found			
215	ORF199	-1	123467	125038	524	hypothetical protein [Pelagibacteraceae bacterium]	2.00E-171	MBC8421784.1	50
216	ORF200	-3	125035	125889	285	hypothetical protein CBD42_11125 [Gammaproteobacteria bacterium TMED182]	3.00E-12	OUW43991.1	27
217	ORF201	-3	125998	126849	284	hypothetical protein [Streptomyces sp. FXJ1.172]	4.00E-12	WP_067055536.1	24
218	ORF202	-2	126846	128228	461	hypothetical protein [Bdellovibrio exovorus]	0	WP_015471054.1	61
219	ORF203	-1	128198	129043	282	hypothetical protein sccontig008-79 [Streptomyces chromofuscus]	4.00E-19	AEZ64587.1	32
220	ORF204	-2	129120	130403	428	hypothetical protein [Burkholderia territorii]	2.00E-67	WP_081081049.1	35
221						ATP-dependent DNA ligase [Bacillus phage SP-10]	2.00E-38	YP_007003455.1	31
222	ORF205	+	129342	130412	357	No significant similarity found			
223	ORF206	2	130451	131167	239	No significant similarity found			
224	ORF207	3	131136	131564	143	hypothetical protein E4H12_11805 [Candidatus Thorarchaeota archaeon]	1.00E-18	TFG96110.1	32
225	ORF208	-2	131565	132578	338	hypothetical protein NVP1081O_160 [Vibrio phage 1.081.O._10N.286.52.C2]	5.00E-52	AUR85895.1	36
226	ORF209	-1	132575	133237	221	hypothetical protein [Paenibacillus riograndensis]	9.00E-05	WP_060862895.1	22
227	ORF210	-2	133294	133875	194	hypothetical protein CBD16_07415 [Betaproteobacteria bacterium TMED156]	1.00E-41	OUV99851.1	43
228						hypothetical protein PBI_121Q_16 [Escherichia phage 121Q]	2.00E-30	YP_009101610.1	41
229	ORF211	-2	134283	134969	229	hypothetical protein CTY12_04910 [Methylotenera sp.]	7.00E-48	PPD53424.1	36
230						hypothetical protein PBI_121Q_14 [Escherichia phage 121Q]	1.00E-09	YP_009101608.1	21
231	ORF212	-3	134971	135426	152	predicted ORF [Xanthomonas phage XacN1]	4.00E-22	BBA65387.1	36
232	ORF213	-2	135426	136346	307	hypothetical protein DRJ15_13365 [Bacteroidetes bacterium]	8.00E-93	RLD77522.1	46
233	ORF214	-	136362	137564	401	DUF2934 domain-containing protein [Propionivibrio sp.]	1.00E-67	MBL8414427.1	41
234	ORF215	-	137561	137875	105	predicted ORF [Xanthomonas phage XacN1]	2.00E-12	BBA65391.1	37
235	ORF216	-	138064	138585	174	DNA polymerase III alpha subunit [Xanthomonas phage XacN1]	9.00E-32	BBA65393.1	39
236	ORF217	-	138548	139012	155	CMP deaminase [Mariniphaga anaerophila]	1.00E-44	WP_073001946.1	52

	A	B	C	D	E	F	G	H	I
237	ORF218	-	139012	139230	73	MULTISPECIES: hypothetical protein [unclassified Nitrosomonadales]	9.00E-07	WP_048356701.1	59
238	ORF219	-	139199	140653	485	No significant similarity			
239	ORF220	-	140654	141028	125	No significant similarity			
240	ORF221	-	140997	141518	174	No significant similarity			
241	ORF222	-	141515	142111	199	alpha/beta fold hydrolase [Verrucomicrobiaceae bacterium]	1.00E-32	RYD62074.1	39
242	ORF223	-	142171	142503	111	thioredoxin [Crenothrix sp. D3]	1.00E-25	OTE97860.1	69
243	ORF224	-	142513	142797	95	No significant similarity			
244	ORF225	-	142794	143939	382	ribonucleotide-diphosphate reductase subunit beta [Shewanella colwelliana]	3.00E-141	WP_028763880.1	54
245	ORF226	-	144040	146370	777	ribonucleoside-diphosphate reductase subunit alpha [Comamonas sp. B-9]	0	WP_027011154.1	51
246	ORF227	-	146461	147042	194	peptidoglycan-binding protein [gamma proteobacterium endosymbiont of Lamellibrachia anaximandri]	4.00E-35	MBL3601609.1	44
247						peptidoglycan-binding protein [Vibrio cholerae]	2.00E-32	OAB93777.1	42
248	ORF228	-	147097	147360	88	MULTISPECIES: DUF4326 domain-containing protein [Thioalkalivibrio]	1.00E-31	WP_013006599.1	57
249						T4 NrdA.1-like protein [Burkholderia phage BcepNazgul]	1.00E-28	NP_919003.1	54
250	ORF229	-	147360	147815	152	No significant similarity found.	2.00E-05	OUV99855.1	47
251	ORF230	-	147819	148085	89	No significant similarity found			
252	ORF231	-	148095	148619	175	hypothetical protein E4H12_10225 [Candidatus Thorarchaeota archaeon]	3.00E-29	TFG96789.1	37
253	ORF232	-	148640	148927	96	No significant similarity found			
254	ORF233	-	148917	149159	81	No significant similarity found			
255	ORF234	-	149131	150414	428	hypothetical protein [Syntrophomonadaceae bacterium]	1.00E-107	MBT9138148.1	50
256						DNA helicase-like protein [Salicola phage SCTP-2]	7.00E-87	ASV44158.1	37
257						helicase [Klebsiella phage K64-1]	1.00E-86	YP_009153172.1	55
258	ORF235	-	150605	150955	117	No significant similarity found			
259	ORF236	-	151030	151488	153	hypothetical protein [bacterium]	9.00E-08	NBV28697.1	27
260	ORF237	-	151481	152338	286	serine/threonine protein phosphatase 1 [Methylocaldum sp. 175]	1.00E-39	SMF95613.1	37
261	ORF238	-	152371	153429	353	hypothetical protein E4H12_13725 [Candidatus Thorarchaeota archaeon]	4.00E-101	TFG95221.1	47

	A	B	C	D	E	F	G	H	I
262						RecA-like recombination and repair protein [Agrobacterium phage Atu_ph07]	4.00E-44	ASV44705.1	59
263	ORF239	-	153493	154359	289	single-stranded DNA binding protein [Xanthomonas phage XacN1]	4.00E-44	BBA65400.1	40
264	ORF240	+	154580	155296	239	No significant similarity found			
265	ORF241	-	155412	156509	366	No significant similarity found			
266	ORF242	-	156614	157639	342	hypothetical protein [Roseibium sp. RKSG952]	4.00E-174	MTH94910.1	69
267						putative RNA ligase [Acinetobacter phage vB_AbaM_ME3]	1.00E-106	AND75422.1	49
268	ORF243	-	157681	157863	61	No significant similarity found			
269	ORF244	-	157932	158216	95	No significant similarity found			
270	ORF245	-	158213	158725	171	putative tRNA ligase/uridine kinase [Bodo saltans virus]	1.00E-24	ATZ80229.1	38
271	ORF246	-	158722	159273	184	No significant similarity found			
272	ORF247	-	159270	159737	156	No significant similarity found			
273	ORF248	-	159734	160165	144	adenylylsulfate kinase [Beijerinckia sp. 28-YEA-48]	8.00E-53	SEB56588.1	57
274						unnamed protein product [Ralstonia phage phiRSL1]	1.00E-23	YP_001950117.1	43
275	ORF249	-	160149	160523	125	MULTISPECIES: hypothetical protein [Sphingomonadaceae]	2.00E-22	WP_007685195.1	42
276	ORF250	-	160611	161426	272	conserved hypothetical protein [Ricinus communis]	7.00E-12	EEF25777.1	45
277	ORF251	-	161484	161897	138	transposase [Burkholderia ubonensis]	3.00E-29	WP_198390660.1	54
278	ORF252	-	162168	162650	161	No significant similarity found			
279	ORF253	+	162753	164330	526	terminase large subunit [Xanthomonas phage XacN1]	7.00E-136	BBA65403.1	41
280	ORF254	-	164332	165075	248	hypothetical protein [Pelagibacter phage HTVC008M]	1.00E-27	YP_007517995.1	32
281	ORF255	-	165072	165797	242	hypothetical protein KQ78_00460 [Candidatus Izimaplasma sp. HR2]	2.00E-12	KFZ27290.1	27
282	ORF256	-	165794	166216	141	No significant similarity found			
283	ORF257	-	166235	166771	179	No significant similarity found			
284	ORF258	-	166817	167473	219	No significant similarity found			
285	ORF259	-	167470	167913	148	hypothetical protein Xoosp13_249 [Xanthomonas phage Xoo-sp13]	3.00E-12	QDJ96435.1	36
286	ORF260	-	167955	168383	143	head completion protein [Synechococcus phage ACG-2014f]	5.00E-37	AIX21328.1	45
287	ORF261	-	168386	169630	415	hypothetical protein [Syntrophomonadaceae bacterium]	2.00E-36	MBT9137697.1	28
288	ORF262	+	169665	170243	193	phospholipase D family protein [Chlorobaculum limnaeum]	1.00E-33	WP_069809568.1	44
289	ORF263	-	170752	171060	103	No significant similarity found			

	A	B	C	D	E	F	G	H	I
290	ORF264	-	171018	171521	168	No significant similarity found			
291	ORF265	-	171523	172137	205	No significant similarity found			
292	ORF266	+	172207	172665	153	hypothetical protein AMJ86_07825 [bacterium SM23_57]	3.00E-19	KPL06626.1	35
293	ORF267	-	172666	173052	129	No significant similarity found			
294	ORF268	+	173085	173681	199	No significant similarity found			
295	ORF269	+	173678	174058	127	No significant similarity found			
296	ORF270	-	173974	174453	160	No significant similarity found			
297	ORF271	+	174077	174772	232	VWA domain-containing protein [Blautia hydrogenotrophica]	8.00E-49	WP_005952142.1	46
298	ORF272	+	174774	175007	78	No significant similarity found			
299	ORF273	+	175012	175335	108	No significant similarity found			
300	ORF274	-	175363	175944	194	No significant similarity found			
301	ORF275	-	175948	176556	203	No significant similarity found			
302	ORF276	-	176603	177223	207	No significant similarity found			
303	ORF277	-	177201	177791	197	No significant similarity found			
304	ORF278	-	177801	178403	201	No significant similarity found			
305	ORF279	-	178400	179032	211	No significant similarity found			
306	ORF280	-	179057	179647	197	No significant similarity found			
307	ORF281	-	179644	180255	204	No significant similarity found			
308	ORF282	-	180252	180827	192	No significant similarity found			
309	ORF283	-	180846	181454	203	No significant similarity found			
310	ORF284	-	181512	182072	187	No significant similarity found			
311	ORF285	-	183205	183441	79	No significant similarity found			
312	ORF286	+	183507	183887	127	No significant similarity found			
313	ORF287	+	183797	184051	85	No significant similarity found			
314	ORF288	-	184325	184552	76	No significant similarity found			
315	ORF289	-	184554	184784	77	No significant similarity found			
316	ORF290	-	184785	185012	76	No significant similarity found			
317	ORF291	-	184996	185238	81	No significant similarity found			
318	ORF292	-	185235	185471	79	No significant similarity found			
319	ORF293	-	185475	185708	78	No significant similarity found			
320	ORF294	-	185705	185998	98	No significant similarity found			

	A	B	C	D	E	F	G	H	I
321	ORF295	-	186138	186953	272	No significant similarity found			
322	ORF296	+	186967	187335	123	hypothetical protein [Acidovorax sp. Root402]	5.00E-12	WP_056058775.1	29
323	ORF297	-	187227	187784	186	No significant similarity found			
324	ORF298	+	187546	187995	150	No significant similarity found			
325	ORF299	+	188008	188220	71	No significant similarity found			
326	ORF300	+	188241	188417	59	No significant similarity found			
327	ORF301	+	188617	189180	188	No significant similarity found			
328	ORF302	+	189177	189758	194	No significant similarity found			
329	ORF303	+	189755	190348	198	No significant similarity found			
330	ORF304	+	190345	190929	195	No significant similarity found			
331	ORF305	+	190926	191483	186	No significant similarity found			
332	ORF306	+	191480	192064	195	No significant similarity found			
333	ORF307	+	192068	192640	191	No significant similarity found			
334	ORF308	+	192707	193309	201	No significant similarity found			
335	ORF309	+	193306	193908	201	No significant similarity found			
336	ORF310	+	193905	194483	193	No significant similarity found			
337	ORF311	+	194480	195049	190	No significant similarity found			
338	ORF312	+	195046	195684	213	No significant similarity found			
339	ORF313	+	195681	196286	202	No significant similarity found			
340	ORF314	+	196283	196900	206	No significant similarity found			
341	ORF315	+	196933	197505	191	No significant similarity found			
342	ORF316	+	197502	198083	194	No significant similarity found			
343	ORF317	+	198080	198673	198	No significant similarity found			
344	ORF318	+	198670	199305	212	No significant similarity found			
345	ORF319	+	199302	199895	198	No significant similarity found			
346	ORF320	-	199898	200485	196	No significant similarity found			
347	ORF321	-	200482	201069	196	No significant similarity found			
348	ORF322	-	201066	201683	206	No significant similarity found			
349	ORF323	+	201864	202133	90	No significant similarity found			
350	ORF324	+	202194	202427	78	No significant similarity found			
351	ORF325	+	202536	203132	199	No significant similarity found			

	A	B	C	D	E	F	G	H	I
352	ORF326	+	203129	203743	205	No significant similarity found			
353	ORF327	+	203762	204415	218	No significant similarity found			
354	ORF328	+	204391	205056	222	No significant similarity found			
355	ORF329	+	205103	205720	206	No significant similarity found			
356	ORF330	+	205717	206328	204	No significant similarity found			
357	ORF331	+	206325	206900	192	No significant similarity found			
358	ORF332	+	206990	207595	202	No significant similarity found			
359	ORF333	-	207872	208144	91	No significant similarity found			
360	ORF334	+	208223	208879	219	No significant similarity found			
361	ORF335	+	208876	209457	194	No significant similarity found			
362	ORF336	+	209454	210056	201	No significant similarity found			
363	ORF337	+	210053	211225	391	No significant similarity found			
364	ORF338	-	211277	211693	139	No significant similarity found			
365	ORF339	-	211722	211997	92	No significant similarity found			
366	ORF340	-	211994	212314	107	No significant similarity found			
367	ORF341	-	212405	212740	112	No significant similarity found			
368	ORF342	+	212829	213419	197	No significant similarity found			
369	ORF343	+	213453	213731	93	No significant similarity found			
370	ORF344	+	213932	214702	257	No significant similarity found			
371	ORF345	-	214749	215081	111	No significant similarity found			
372	ORF346	-	215128	215907	260	No significant similarity found			
373	ORF347	-	215949	216632	228	hypothetical protein [Euryarchaeota archaeon]	4.00E-23	MAV94147.1	35
374	ORF348	-	216629	217330	234	hypothetical protein [Candidatus Marinimicrobia bacterium]	3.00E-64	WP_010606649.1	46
375	ORF349	-	217327	218748	474	radical SAM protein [Bdellovibrionales bacterium]	2.00E-166	NQZ18283.1	51
376	ORF350	-	218735	219688	318	hypothetical protein CBB97_19390 [Candidatus Endolissoclinum sp. TMED37]	4.00E-93	OUU19416.1	46
377	ORF351	-	219681	220235	185	No significant similarity found			
378	ORF352	-	220232	221080	283	hypothetical protein CO117_12630 [Flavobacteriaceae bacterium CG_4_9_14_3_um_filter_33_16]	2.00E-73	PJB17181.1	45
379	ORF353	-	221077	221946	290	hypothetical protein [Euryarchaeota archaeon]	3.00E-57	MBR20724.1	35
380	ORF354	-	221939	222310	124	hypothetical protein [bacterium]	1.00E-16	NBP58664.1	40
381	ORF355	-	222384	223553	390	hypothetical protein [Candidatus Poribacteria bacterium]	4.00E-17	MAJ71405.1	30

	A	B	C	D	E	F	G	H	I
382	ORF356	-	223631	224212	194	structural protein [Xanthomonas phage XacN1]	4.00E-44	BBA65499.1	44
383	ORF357	-	224282	225076	265	No significant similarity found			
384	ORF358	-	225112	226089	326	GDP-mannose 4,6-dehydratase [Enterobacter hormaechei]	1.00E-92	WP_190319761.	47
385	ORF359	-	226174	227745	524	tail sheath protein [Xanthomonas phage XacN1]	8.00E-94	BBA65515.1	48
386	ORF360	-	227849	229036	396	No significant similarity found			
387	ORF361	-	229048	236670	2541	structural protein [Pectobacterium phage CBB]	2.00E-17	AMM43801.1	25
388	ORF362	-	236776	240420	1215	baseplate wedge [Cronobacter phage vB_CsaM_GAP32]	2.00E-93	YP_006987336.1	36
389	ORF363	-	240586	240963	126	baseplate wedge [Xanthomonas phage XacN1]	2.00E-26	BBA65471.1	46
390	ORF364	-	240963	242090	376	baseplate hub subunit and tail lysozyme [Pseudomonas phage vB_PaeM_PA5oct]	2.00E-29	QCG76015.1	30
391	ORF365	-	242080	244293	738	hypothetical protein [Syntrophomonadaceae bacterium]	1.00E-14	MBT9137474.1	27
392	ORF366	-	244293	244664	124	hypothetical protein E4H14_05610 [Candidatus Thorarchaeota archaeon]	5.00E-19	TFH08860.1	48
393	ORF367	-	244672	245745	358	structural protein [Serratia phage BF]	2.00E-17	AQW88767.1	30
394	ORF368	-	245738	246349	204	Nicotinamide-nucleotide adenylyltransferase [Syntrophomonadaceae bacterium]	2.00E-31	MBT9137471.1	33
395	ORF369	+	246521	247264	248	hypothetical protein CBE14_02005 [Rickettsiales bacterium TMED254]	9.00E-23	OUX28910.1	49
396						sigma factor for late transcription [Agrobacterium phage Atu_ph07]	3.00E-19	ASV44763.1	47
397	ORF370	+	247266	248333	356	hypothetical protein CBB97_04730 [Candidatus Endolissoclinum sp. TMED37]	2.00E-65	OUU28347.1	37
398						recombination endonuclease [Salicola phage SCTP-2]	2.00E-52	ASV44555.1	33
399	ORF371	+	248317	250059	581	endonuclease subunit [uncultured Caudovirales phage]	2.00E-87	CAB4241474.1	32
400	ORF372	+	250170	250727	186	hypothetical protein E6R13_07855 [Spirochaetes bacterium	7.00E-19	TXG80555.1	34
401	ORF373	+	250724	251365	214	No significant similarity found			
402	ORF374	+	251366	251623	86	No significant similarity found			
403	ORF375	-	253025	253825	267	baseplate hub subunit [Agrobacterium phage Atu_ph07]	2.00E-23	ASV44759.1	28
404	ORF376	+	253913	256468	852	No significant similarity found			
405	ORF377	-	256471	257010	180	No significant similarity found			
406	ORF378	+	257102	258826	575	portal vertex protein [Xanthomonas phage XacN1]	4.00E-133	BBA65449.1	42
407						portal protein [Salicola phage SCTP-2]	1.00E-92	ASV44105.1	35

	A	B	C	D	E	F	G	H	I
408	ORF379	+	259178	259705	176	prohead core scaffolding protein and protease [Xanthomonas phage XacN1]	1.00E-51	BBA65445.1	51
409	ORF380	+	259705	260019	105	No significant similarity found			
410	ORF381	+	260019	260246	76	No significant similarity found			
411	ORF382	+	260369	261238	290	hypothetical protein E4H14_06940 [Candidatus Thorarchaeota archaeon]	6.00E-61	TFH08171.1	44
412						hypothetical protein PBI_SCTP2_94 [Salicola phage SCTP-2]	3.00E-14	ASV44109.1	31
413	ORF383	+	261286	262521	412	major capsid protein [Salicola phage SCTP-2]	7.00E-104	ASV44110.1	42
414	ORF384	+	262617	263249	211	hypothetical protein SmphiM12_106 [Sinorhizobium phage phiM12]	1.00E-08	YP_009143006.1	47
415	ORF385	-	263233	263730	166	hypothetical protein UFOVP71_345 [uncultured Caudovirales phage]	1.00E-22	CAB4241807.1	42
416	ORF386	-	263793	264287	165	No significant similarity found			
417	ORF387	+	263829	264308	160	hypothetical protein [Salmonella enterica	3.00E-15	ECQ2932879.1	33
418	ORF388	+	264321	264602	94	No significant similarity found			
419	ORF389	-	264297	264674	126	No significant similarity found			
420	ORF390	+	264556	265020	155	hypothetical protein [Pusillimonas noertemannii]	5.00E-21	WP_116517675.1	45
421	ORF391	-	264996	265478	161	No significant similarity found			
422	ORF392	+	265033	265563	177	hypothetical protein [Cronobacter malonaticus]	2.00E-17	WP_032967867.1	33
423						hypothetical protein AMBK_46 [Salmonella phage vB_SosS_Oslo]	6.00E-13	YP_006560853.1	30
424	ORF393	+	265564	265770	69	No significant similarity found			
425	ORF394	+	265770	266228	153	thymidylate kinase [Caudovirales sp. ctOwN3]	5.00E-23	QGH72154.1	37
426	ORF395	+	266212	266472	87	No significant similarity found			
427	ORF396	+	266484	266705	74	hypothetical protein [Candidatus Sericytochromatia bacterium]	7.00E-45	AVH85324.1	84
428	ORF397	+	266707	267747	347	RNA ligase [Caulobacter phage Cr30]	5.00E-98	YP_009098789.1	47
429						RnlB RNA ligase 2 [Ralstonia phage RSP15	8.00E-87	YP_009277122.1	44
430	ORF398	+	267747	267971	75	No significant similarity found			
431	ORF399	+	267982	268245	88	hypothetical protein [Pseudomonas phage phiPsa374]	6.00E-26	YP_009009411.1	63
432						von Willebrand factor type A domain protein [Pseudomonas phage VCM]	6.00E-07	YP_009222660.1	43

	A	B	C	D	E	F	G	H	I
433	ORF400	+	268295	268480	62	No significant similarity found			
434	ORF401	+	268348	269829	494	hypothetical protein [Syntrophomonadaceae bacterium]	6.00E-84	MBT9138137.1	41
435						neck protein [Pectobacterium phage CBB]	2.00E-23	AMM43766.1	30
436	ORF402	+	269836	271368	511	hypothetical protein [Xanthomonas phage XacN1]	1.00E-100	BBA65415.1	38
437	ORF403	+	271358	272233	292	hypothetical protein KQ78_00461 [Candidatus Izimaplasma sp. HR2]	3.00E-13	KFZ27291.1	24
438						hypothetical protein PBI_SCTP2_385 [Salicola phage SCTP-2]	4.00E-10	ASV44400.1	35
439	ORF404	+	272220	273137	306	hypothetical protein KQ78_00461 [Candidatus Izimaplasma sp. HR2]	9.00E-09	KFZ27291.1	24
440						hypothetical protein PBI_SCTP2_385 [Salicola phage SCTP-2]	1.00E-06	ASV44400.1	27
441	ORF405	+	273134	274009	292	hypothetical protein [Agrobacterium phage Atu_ph07]	6.00E-12	YP_009611723.1	37
442						hypothetical protein PBI_SCTP2_385 [Salicola phage SCTP-2]	1.00E-06	ASV44400.1	27
443	ORF406	+	274017	274829	271	hypothetical protein [Syntrophomonadaceae bacterium]	3.00E-82	MBT9137706.1	50
444						proximal tail sheath stabilization [uncultured Mediterranean phage uvMED]	7.00E-22	BAR27635.1	28
445	ORF407	+	274846	277170	775	hypothetical protein [Ralstonia solanacearum]	5.00E-13	WP_087452134.1	39
446	ORF408	+	277191	277904	238	tail fiber domain-containing protein [Salmonella enterica subsp. enterica serovar Typhi]	8.00E-10	CHE41569.1	47
447	ORF409	+	277917	278306	130	No significant similarity found			
448	ORF410	+	278320	279018	233	putative tail fiber protein [Pseudomonas phage Noxifer]	2.00E-07	ARV77307.1	46
449	ORF411	+	279020	281929	970	unnamed protein product [Ralstonia phage phiRSL1]	0	YP_001950068.1	55
450						hypothetical protein [Ralstonia phage RSP15]	0	YP_009276965.1	48
451	ORF412	+	281940	282188	83	unnamed protein product [Ralstonia phage phiRSL1]	6.00E-20	YP_001950069.1	59
452	ORF413	+	282240	282818	193	hypothetical protein [Euryarchaeota archaeon]	6.00E-39	MBR19713.1	45
453						hypothetical protein GAP32_224 [Cronobacter phage vB_CsaM_GAP32]	5.00E-27	YP_006987329.1	38
454						structural protein [Pectobacterium phage CBB]	2.00E-26	AMM43795.1	38
455	ORF414	-	282988	283323	112	No significant similarity found			
456	ORF415	+	283386	285101	572	phage-related tail fiber protein [uncultured Mediterranean phage uvMED]	1.00E-29	BAR25957.1	33
457	ORF416	+	285114	288887	1258	unnamed protein product [Ralstonia phage phiRSL1]	0	YP_001950071.1	73

	A	B	C	D	E	F	G	H	I
458	ORF417	+	288923	289783	287	hypothetical protein [Candidatus Poribacteria bacterium]	2.00E-21	MAJ71405.1	30
459	ORF418	+	289857	293966	1370	unnamed protein product [Ralstonia phage phiRSL1]	0	YP_001950060.1	52
460						putative long tail fiber protein p37, partial [Escherichia coli 1-182-04_S1_C3]	3.00E-21	EZJ96898.1	40
461	ORF419	+	293977	296217	747	unnamed protein product [Ralstonia phage phiRSL1]	0	YP_001950062.1	79
462	ORF420	+	296219	296431	71	unnamed protein product [Ralstonia phage phiRSL1]	2.00E-09	YP_001950064.1	53
463	ORF421	+	296442	298640	733	unnamed protein product [Ralstonia phage phiRSL1]	0	YP_001950063.1	72
464	ORF422	+	298702	299025	108	No significant similarity found			
465	ORF423	+	299043	299432	130	No significant similarity found			
466	ORF424	+	299422	299739	106	No significant similarity found			
467	ORF425	+	299739	301409	557	Murein DD-endopeptidase MepM [Syntrophomonadaceae bacterium]	8.00E-61	MBT9137488.1	34
468	ORF426	+	301412	301699	96	PaaR repeat-containing protein [Ruegeria mobilis]	8.00E-10	WP_074712575.1	47
469	ORF427	+	301699	302592	298	hypothetical protein E4H14_06735 [Candidatus Thorarchaeota archaeon]	6.00E-05	TFH08279.1	50
470	ORF428	-	302737	304050	438	No significant similarity found			
471	ORF429	+	302776	304161	462	hypothetical protein [Proteobacteria bacterium]	4.00E-57	NBP02942.1	53
472	ORF430	-	304213	304485	91	MULTISPECIES: co-chaperone GroES [Ralstonia]	2.00E-08	WP_013213355.1	33
473	ORF431	-	304644	305294	217	hypothetical protein [Syntrophomonadaceae bacterium]	1.00E-51	MBT9137508.1	46
474	ORF432	-	305304	305846	181	No significant similarity found			
475	ORF433	-	305901	306614	238	hypothetical protein BN7874_122 [Phage NCTB]	4.00E-04	SBV38291.1	21
476	ORF434	-	306617	307351	245	No significant similarity found			
477	ORF435	-	307406	308074	223	putative exonuclease [Serratia phage phiMAM1]	6.00E-40	YP_007349054.1	39
478	ORF436	-	308071	308292	74	No significant similarity found			
479	ORF437	-	308289	309449	387	hypothetical protein [Syntrophomonadaceae bacterium]	4.00E-13	MBT9137513.1	47
480	ORF438	-	309479	311161	561	uncharacterized protein PA5oct_304 [Pseudomonas phage vB_PaeM_PA5oct]	3.00E-21	AWN03025.1	43
481	ORF439	-	311220	311597	126	No significant similarity found			
482	ORF440	-	311600	311926	109	No significant similarity found			

	A	B	C	D	E	F	G	H	I
483	ORF441	-	312188	313516	443	DNA primase-helicase subunit [Agrobacterium phage Atu_ph07]	4.00E-95	ASV44692.1	42
484	ORF442	-	313594	314265	224	hydrolase [Pseudomonas sp. MIACH]	2.00E-08	WP_053139728.1	35
485	ORF443	-	314262	315233	324	hypothetical protein CBC91_06285 [Rickettsiales bacterium TMED131]	1.00E-25	OUV76456.1	30
486						DNA primase subunit [Agrobacterium phage Atu_ph07]	9.00E-20	ASV44693.1	27
487	ORF444	-	315302	315991	230	DNA primase [Syntrophomonadaceae bacterium]	1.00E-45	MBT9137518.1	33
488	ORF445	-	316066	317973	636	peptidase [uncultured Mediterranean phage uvMED]	3.00E-25	BAR35475.1	42
489	ORF446	-	317963	319018	352	CobS [Synechococcus phage S-WAM2]	8.00E-49	YP_009324303.1	35
490	ORF447	-	319117	319527	137	hypothetical protein [Syntrophomonadaceae bacterium]	8.00E-23	MBT9137521.1	50
491	ORF448	-	319603	320598	332	DNA polymerase I [Syntrophomonadaceae bacterium]	3.00E-93	AVH85376.1	44
492						DNA polymerase I [Escherichia phage vB_Eco_slurp01]	7.00E-25	SCA80493.1	31
493	ORF449	-	320601	320903	101	No significant similarity found			
494	ORF450	-	320906	321628	241	hypothetical protein CTY12_00330 [Methylotenera sp.]	1.00E-16	PPD55738.1	27
495	ORF451	-	321631	324054	808	DNA polymerase [Xanthomonas phage XacN1]	8.00E-167	BBA65491.1	37
496	ORF452	+	324213	324422	70	No significant similarity found			
497	ORF453	-	324331	324603	91	double-stranded DNA binding protein [Vibrio phage vB_VmeM-32]	4.00E-05	ALY07093.1	38
498	ORF454	-	324614	325012	133	No significant similarity found			
499	ORF455	-	325019	325423	135	No significant similarity found			
500	ORF456	-	325423	326460	346	No significant similarity found			
501	ORF457	-	326499	326906	136	No significant similarity found			
502	ORF458	-	326884	327468	195	DNA polymerase III epsilon subunit [Xanthomonas phage XacN1]	3.00E-32	BBA65371.1	41
503						3'-5' exonuclease [Xenorhabdus bovienii]	2.00E-06	WP_038193281.1	26
504	ORF459	-	327465	328040	192	ATP-binding protein [Vibrio gigantis]	9.00E-05	WP_086714681.1	25
505	ORF460	+	327986	328279	98	No significant similarity found			
506	ORF461	-	328037	328306	90	No significant similarity found			
507	ORF462	-	328390	329211	274	phoH family protein [Corallococcus sp. CAG:1435]	1.00E-51	CCZ95472.1	45
508	ORF463	-	329145	329495	117	No significant similarity found			
509	ORF464	-	329497	330060	188	No significant similarity found			
510	ORF465	-	330082	330747	222	hypothetical protein CBE27_01000 [Pelagibacteraceae bacterium TMED267]	5.00E-36	OUX65662.1	39

	A	B	C	D	E	F	G	H	I
511						ATP-dependent Clp protease proteolytic subunit [Geobacter anodireducens]	4.00E-23	WP_082833093.1	34
512	ORF466	-	330768	331049	94	No significant similarity found			
513	ORF467	-	331052	331591	180	No significant similarity found			
514	ORF468	-	331927	332181	85	No significant similarity found			
515	ORF469	-	332241	332456	72	No significant similarity found			
516	ORF470	-	332504	333007	168	macro domain-containing protein [Paraburkholderia sp. UCT31]	2.00E-35	WP_187631037.1	49
517	ORF471	-	333004	333492	163	No significant similarity found			
518	ORF472	-	333449	334240	264	No significant similarity found			
519	ORF473	-	334304	335233	310	Td thymidylate synthetase [Acinetobacter phage Acj61]	3.00E-167	YP_004009838.1	74
520	ORF474	-	335205	335498	98	No significant similarity found			
521	ORF475	-	335449	335664	72	No significant similarity found			
522	ORF476	-	335661	336026	122	hypothetical protein [Vibrio maritimus]	3.00E-06	WP_042472468.1	43
523	ORF477	-	336023	336469	149	NTP-PPase [Caudovirales sp. ctOwN3]	3.00E-32	QGH72159.1	36
524	ORF478	-	336497	336742	82	No significant similarity found			
525	ORF479	-	336750	336965	72	No significant similarity found			
526	ORF480	-	336962	338041	360	2'-5' RNA ligase [Paraburkholderia sp. C35]	2.00E-84	WP_109482880.1	41
527	ORF481	-	338083	338424	114	hypothetical protein A3K78_05175 [Candidatus Bathyarchaeota archaeon RBG_13_52_12]	4.00E-15	OGD56555.1	43
528	ORF482	-	338425	339138	238	hypothetical protein E4H14_09395 [Candidatus Thorarchaeota archaeon]	5.00E-75	TFH07051.1	50
529	ORF483	-	339307	340632	442	DNA gyrase subunit A [Syntrophomonadaceae bacterium]	1.00E-128	MBT9137648.1	45
530						DNA topoisomerase II medium subunit [Xanthomonas phage XacN1]	2.00E-89	BBA65330.1	38
531	ORF484	-	340683	341063	127	hypothetical protein [Bacillus thuringiensis]	6.00E-09	WP_088011104.1	38
532						hypothetical protein SCRM01_224 [Synechococcus phage S-CRM01]	3.00E-07	YP_004508657.1	38
533	ORF485	-	341063	342979	639	GyrB Type IIA topoisomerase (DNA gyrase/topo II, topoisomerase IV), B subunit [uncultured Caudovirales phage]	0.00E+00	CAB4159554.1	46
534	ORF486	-	343044	343793	250	bifunctional protein GlmU [bacterium BMS3Bbin11]	8.00E-06	GBE46073.1	24
