# Supplementary material for: Biological and Molecular Characterization of a Jumbo Bacteriophage Infecting Plant Pathogenic Ralstonia solanacearum Species Complex Strains
Source: Front Microbiol. 2021 Sep 27;12:741600. doi: 10.3389/fmicb.2021.741600 (PMC8504454; doi:10.3389/fmicb.2021.741600)
Supplement: Supplementary file 1 [file Data_Sheet_1.zip › Supplementary Table S4.PDF]

Supplementary Table S4. Orthologous Average Nucleotide Identity (OrthoANI) values between RsoM2USA and other 46 Jumbo phages in the family of Myoviridae.

	**Ralstonia phage RsoM2USA**	*Achromobacter virus Motura*	*Acinetobacter virus ME3*	*Aeromonas virus 65*	*Agrobacterium virus Atuph07*	*Bacillus virus G*	*Bacillus virus PBS1*	*Bacillus virus SP15*	*Burkholderia virus BcepSaruman*	*Cronobacter virus GAP32*	*Dickeya virus JA11*	*Edwardsiella virus pEtSU*	*Erwinia virus Alexandra*	*Erwinia virus Asesino*	*Erwinia virus Derbicus*	*Erwinia virus Deimos*	*Erwinia virus EaH1*	*Erwinia virus Machinia*	*Erwinia virus Risingsun*	*Erwinia virus Wellington*	*Erwinia virus Y3*	*Erwinia virus Yoloswag*	*Escherichia virus 121Q*	*Escherichia virus Goslar*	*Klebsiella virus K64-1*	*Prochlorococcus virus PSSM2*	*Pseudomonas virus EL*	*Pseudomonas virus Noxifer*	*Pseudomonas virus PaBG*	*Pseudomonas virus phiKZ*	*Ralstonia virus RP12*	*Ralstonia virus RP31*	*Ralstonia virus RSF1*	*Ralstonia virus RSL1*	*Ralstonia virus RSL2*	*Salmonella virus SPN3US*	Serratia phage PCH45	*Serratia virus BF*	*Synechococcus virus ACG2014f*	*Synechococcus virus Bellamy*	*Synechococcus virus SSKS1*	*Tenacibaculum virus pT24*	*Tenacibaculum virus pTm1*	*Vibrio virus Aphrodite1*	*Vibrio virus KVP40*	*Vibrio virus pTD1*	Xanthomonas phage XacN1
**Ralstonia phage RsoM2USA**	**-**	**0**	**57.82**	**61.04**	**0**	**0**	**0**	**0**	**0**	**59.57**	**0**	**62.72**	**0**	**0**	**0**	**0**	**0**	**0**	**0**	**0**	**0**	**0**	**60.28**	**0**	**58.66**	**57.14**	**0**	**0**	**0**	**0**	**0**	**0**	**0**	**63.28**	**0**	**0**	**0**	**58.39**	**0**	**56.16**	**60.10**	**0**	**57.71**	**0**	**58.97**	**0**	**59.29**
Achromobacter virus Motura	**0**	-	0	0	0	0	0	0	62.74	0	58.93	0	60.60	0	0	0	0	0	0	0	60.42	58.56	0	0	0	0	0	0	63.84	0	0	0	0	61.70	0	0	0	0	0	0	0	0	0	0	63.27	0	0
Acinetobacter virus ME3	**57.82**	0	-	0	0	0	0	0	0	0	0	0	0	0	0	0	0	0	0	0	0	0	0	0	0	0	0	0	0	0	0	0	0	0	0	0	0	0	0	0	0	0	0	0	0	0	0
Aeromonas virus 65	**61.04**	0	0	-	0	0	0	0	0	60.95	0	58.81	0	0	0	0	0	0	0	0	0	0	59.04	0	58.56	58.46	0	0	0	0	55.83	56.05	0	0	0	0	0	60.52	0	0	0	0	68.75	0	62.01	0	59.47
Agrobacterium virus Atuph07	**0**	0	0	0	-	60.66	0	0	0	61.04	0	0	0	0	0	0	0	0	0	0	0	0	60.72	0	60.90	0	0	0	0	0	0	0	0	0	62.01	0	0	59.85	0	0	0	0	0	0	0	0	0
Bacillus virus G	**0**	0	0	0	60.66	-	65.42	60.50	0	0	0	0	0	0	0	0	0	0	0	0	0	0	0	0	0	0	0	0	0	0	0	0	0	0	0	0	0	0	0	0	0	0	0	0	0	0	0
Bacillus virus PBS1	**0**	0	0	0	0	65.42	-	0	0	0	0	0	0	0	0	0	0	0	0	0	0	0	0	0	0	0	0	0	0	0	0	0	0	0	0	0	0	0	0	0	0	0	0	0	0	0	0
Bacillus virus SP15	**0**	0	0	0	0	60.50	0	-	0	0	0	0	0	0	0	0	0	0	0	0	0	0	0	0	0	0	0	0	0	0	0	0	0	0	0	0	0	0	0	0	0	0	0	0	0	0	0
Burkholderia virus BcepSaruman	**0**	62.74	0	0	0	0	0	0	-	0	0	0	0	0	0	0	0	0	0	0	0	0	0	0	0	0	0	0	59.87	0	64.68	0	0	64.85	0	0	0	0	0	0	0	0	0	0	0	0	0
Cronobacter virus GAP32	**59.57**	0	0	60.95	61.04	0	0	0	0	-	0	60.04	0	0	0	0	0	0	0	0	0	0	67.48	0	66.72	56.47	0	0	0	0	0	0	0	0	0	0	0	75.34	62.30	59.26	58.19	0	56.52	0	59.03	0	0
Dickeya virus JA11	**0**	58.93	0	0	0	0	0	0	0	0	-	0	63.32	0	0	0	0	0	0	0	64.56	65.95	0	0	0	0	0	0	61.42	0	0	0	0	0	0	0	0	0	0	0	0	0	0	0	0	0	0
Edwardsiella virus pEtSU	**62.72**	0	0	58.81	0	0	0	0	0	60.04	0	-	0	0	61.48	67.29	0	0	59.46	0	0	0	60.25	0	0	0	58.13	0	0	0	0	0	0	0	0	0	75.11	60.00	59.21	0	0	0	0	0	0	59.22	0
Erwinia virus Alexandra	**0**	60.60	0	0	0	0	0	0	0	0	63.32	0	-	0	0	0	0	0	0	0	73.22	65.95	0	0	0	0	0	0	0	0	0	0	0	56.78	0	0	0	0	0	0	0	0	0	0	0	0	0
Erwinia virus Asesino	**0**	0	0	0	0	0	0	0	0	0	0	0	0	-	64.02	58.96	58.22	67.19	0	67.07	0	0	0	0	0	0	0	0	0	0	57.29	58.05	0	0	0	70.12	0	0	0	0	0	0	0	0	0	0	0
Erwinia virus Derbicus	**0**	0	0	0	0	0	0	0	0	0	0	61.48	0	64.02		57.25	67.46	64.05	68.79	63.08	0	0	0	61.05	0	0	0	0	0	0	0	0	0	0	0	63.86	0	0	0	0	0	0	0	0	0	0	0
Erwinia virus Deimos	**0**	0	0	0	0	0	0	0	0	0	0	67.29	0	58.96	57.25	-	64.23	0	58.70	56.13	64.72	0	0	55.21	57.61	0	0	56.35	0	0	61.70	60.20	0	0	0	58.85	0	56.47	58.14	0	0	0	0	0	59.85	0	0
Erwinia virus EaH1	**0**	0	0	0	0	0	0	0	0	0	0	0	0	58.22	67.46	64.23	-	55.92	65.44	64.23	0	0	0	0	0	0	0	60.76	0	0	56.33	61.45	0	0	0	0	61.86	0	0	0	0	0	0	0	0	0	0
Erwinia virus Machinia	**0**	0	0	0	0	0	0	0	0	0	0	0	0	67.19	64.05	0	55.92	-	0	66.25	0	0	0	63.39	0	0	0	0	0	0	0	0	0	0	0	67.06	0	0	0	0	0	0	0	0	0	0	0
Erwinia virus Risingsun	**0**	0	0	0	0	0	0	0	0	0	0	59.46	0	0	68.79	58.70	65.44	0	-	0	0	0	0	0	0	0	62.19	0	0	0	0	0	0	0	0	0	0	0	0	0	0	0	0	59.72	0	60.67	0
Erwinia virus Wellington	**0**	0	0	0	0	0	0	0	0	0	0	0	0	67.07	63.08	56.13	64.23	66.25	0	-	0	0	0	61.23	0	0	0	0	0	0	0	59.00	0	0	0	0	0	0	0	0	0	0	0	0	0	0	0
Erwinia virus Y3	**0**	60.42	0	0	0	0	0	0	0	0	64.56	0	73.22	0	0	64.72	0	0	0	0	-	66.45	0	0	0	0	0	0	61.10	0	0	0	0	0	0	0	0	0	0	0	0	0	0	0	0	0	0
Erwinia virus Yoloswag	**0**	58.56	0	0	0	0	0	0	0	0	65.95	0	65.95	0	0	0	0	0	0	0	66.45	-	0	0	0	0	0	0	61.60	0	0	0	0	0	0	0	0	0	0	0	0	0	0	0	0	0	0
Escherichia virus 121Q	**60.28**	0	0	59.04	60.72	0	0	0	0	67.48	0	60.25	0	0	0	0	0	0	0	0	0	0	-	0	70.25	62.44	0	0	0	0	0	0	0	0	0	0	0	67.95	60.17	57.80	57.35	0	57.48	0	58.93	0	0
Escherichia virus Goslar	**0**	0	0	0	0	0	0	0	0	0	0	0	0	0	61.05	55.21	0	63.39	0	61.23	0	0	0	-	0	0	0	0	0	0	0	57.58	0	0	0	60.07	60.35	0	0	0	0	0	0	0	0	0	0
Klebsiella virus K64-1	**58.66**	0	0	58.56	60.90	0	0	0	0	66.72	0	0	0	0	0	57.61	0	0	0	0	0	0	70.25	0	-	57.68	0	0	0	0	0	0	0	0	0	0	0	66.65	58.67	61.01	58.67	0	57.42	0	59.24	0	0
Prochlorococcus virus PSSM2	**57.14**	0	0	58.46	0	0	0	0	0	56.47	0	0	0	0	0	0	0	0	0	0	0	0	62.44	0	57.68	-	0	0	0	0	0	0	0	0	0	0	0	60.58	66.46	67.81	69.21	0	0	0	59.16	0	0
Pseudomonas virus EL	**0**	0	0	0	0	0	0	0	0	0	0	58.13	0	0	0	0	0	0	62.19	0	0	0	0	0	0	0	-	0	0	0	0	0	0	0	0	0	0	0	0	0	0	0	0	59.24	0	58.38	0
Pseudomonas virus Noxifer	**0**	0	0	0	0	0	0	0	0	0	0	0	0	0	0	56.35	60.76	0	0	0	0	0	0	0	0	0	0	-	0	58.90	58.05	58.56	55.96	0	58.94	0	59.21	0	0	0	0	0	0	0	0	0	0
Pseudomonas virus PaBG	**0**	63.84	0	0	0	0	0	0	59.87	0	61.42	0	0	0	0	0	0	0	0	0	61.10	61.60	0	0	0	0	0	0	-	0	0	0	0	60.58	0	0	0	0	0	0	0	0	0	0	0	0	0
Pseudomonas virus phiKZ	**0**	0	0	0	0	0	0	0	0	0	0	0	0	0	0	0	0	0	0	0	0	0	0	0	0	0	0	58.90	0	-	64.31	60.93	0	0	0	57.48	0	0	0	0	0	0	0	0	0	0	0
Ralstonia virus RP12	**0**	0	0	55.83	0	0	0	0	64.68	0	0	0	0	57.29	0	61.70	56.33	0	0	0	0	0	0	0	0	0	0	58.05	0	64.31	-	98.73	59.07	0	58.59	0	57.69	0	0	0	0	0	0	0	0	0	0
Ralstonia virus RP31	**0**	0	0	56.05	0	0	0	0	0	0	0	0	0	58.05	0	60.20	61.45	0	0	59.00	0	0	0	57.58	0	0	0	58.56	0	60.93	98.73	-	59.53	61.21	58.70	0	59.34	0	0	0	0	0	0	0	0	0	0
Ralstonia virus RSF1	**0**	0	0	0	0	0	0	0	0	0	0	0	0	0	0	0	0	0	0	0	0	0	0	0	0	0	0	55.96	0	0	59.07	59.53	-	0	79.50	0	0	0	0	0	0	0	0	0	0	0	0
Ralstonia virus RSL1	**63.28**	61.70	0	0	0	0	0	0	64.85	0	0	0	56.78	0	0	0	0	0	0	0	0	0	0	0	0	0	0	0	60.58	0	0	61.21	0	-	0	0	0	0	0	0	0	0	0	0	0	0	0
Ralstonia virus RSL2	**0**	0	0	0	62.01	0	0	0	0	0	0	0	0	0	0	0	0	0	0	0	0	0	0	0	0	0	0	58.94	0	0	58.59	58.70	79.50	0	-	0	0	0	0	0	0	0	0	0	0	0	0
Salmonella virus SPN3US	**0**	0	0	0	0	0	0	0	0	0	0	0	0	70.12	63.86	58.85	0	67.06	0	0	0	0	0	60.07	0	0	0	0	0	57.48	0	0	0	0	0	-	0	0	0	0	0	0	0	0	0	0	0
Serratia phage PCH45	**0**	0	0	0	0	0	0	0	0	0	0	75.11	0	0	0	0	61.86	0	0	0	0	0	0	60.35	0	0	0	59.21	0	0	57.69	59.34	0	0	0	0	-	57.80	59.51	59.91	0	0	0	0	0	0	57.12
Serratia virus BF	**58.39**	0	0	60.52	59.85	0	0	0	0	75.34	0	60.00	0	0	0	56.47	0	0	0	0	0	0	67.95	0	66.65	60.58	0	0	0	0	0	0	0	0	0	0	57.80	-	57.61	60.38	58.96	0	55.24	0	59.20	0	0
Synechococcus virus ACG2014f	**0**	0	0	0	0	0	0	0	0	62.30	0	59.21	0	0	0	58.14	0	0	0	0	0	0	60.17	0	58.67	66.46	0	0	0	0	0	0	0	0	0	0	59.51	57.61	-	68.15	67.36	0	0	0	58.72	0	0
Synechococcus virus Bellamy	**56.16**	0	0	0	0	0	0	0	0	59.26	0	0	0	0	0	0	0	0	0	0	0	0	57.80	0	61.01	67.81	0	0	0	0	0	0	0	0	0	0	59.91	60.38	68.15	-	70.79	0	0	0	58.77	0	0
Synechococcus virus SSKS1	**60.10**	0	0	0	0	0	0	0	0	58.19	0	0	0	0	0	0	0	0	0	0	0	0	57.35	0	58.67	69.21	0	0	0	0	0	0	0	0	0	0	0	58.96	67.36	70.79	-	0	58.29	0	0	0	0
Tenacibaculum virus pT24	**0**	0	0	0	0	0	0	0	0	0	0	0	0	0	0	0	0	0	0	0	0	0	0	0	0	0	0	0	0	0	0	0	0	0	0	0	0	0	0	0	0	-	64.18	0	0	0	0
Tenacibaculum virus pTm1	**57.71**	0	0	68.75	0	0	0	0	0	56.52	0	0	0	0	0	0	0	0	0	0	0	0	57.48	0	57.42	0	0	0	0	0	0	0	0	0	0	0	0	55.24	0	0	58.29	64.18	-	0	0	0	0
Vibrio virus Aphrodite1	**0**	0	0	0	0	0	0	0	0	0	0	0	0	0	0	0	0	0	59.72	0	0	0	0	0	0	0	59.24	0	0	0	0	0	0	0	0	0	0	0	0	0	0	0	0	-	0	71.01	0
Vibrio virus KVP40	**58.97**	63.27	0	62.01	0	0	0	0	0	59.03	0	0	0	0	0	59.85	0	0	0	0	0	0	58.93	0	59.24	59.16	0	0	0	0	0	0	0	0	0	0	0	59.20	58.72	58.77	0	0	0	0	-	0	0
Vibrio virus pTD1	**0**	0	0	0	0	0	0	0	0	0	0	59.22	0	0	0	0	0	0	60.67	0	0	0	0	0	0	0	58.38	0	0	0	0	0	0	0	0	0	0	0	0	0	0	0	0	71.01	0	-	0
Xanthomonas phage XacN1	**59.29**	0	0	59.47	0	0	0	0	0	0	0	0	0	0	0	0	0	0	0	0	0	0	0	0	0	0	0	0	0	0	0	0	0	0	0	0	57.12	0	0	0	0	0	0	0	0	0	-
