# Supplementary material for: Biological and Molecular Characterization of a Jumbo Bacteriophage Infecting Plant Pathogenic Ralstonia solanacearum Species Complex Strains
Source: Front Microbiol. 2021 Sep 27;12:741600. doi: 10.3389/fmicb.2021.741600 (PMC8504454; doi:10.3389/fmicb.2021.741600)
Supplement: Supplementary file 1 [file Data_Sheet_1.zip › Supplementary Table S5.PDF]

Supplementary Table S5

Comparison of general characteristics between jumbo phages RsoM2USA and XacN1

General characteristics	Phage Identifier Name	
	RsoM2USA	XacN1
GenBank accession	MG752970	MF360958
Host species	*R. solanacearum*<br>*R. pseudosolanacearum*<br>*R. syzygii*	*Xanthomonas citri*
Host range	wide	wide
Growth cycle (min)	360	240
Latent period (min)	270	90
Burst size (PFU/cell)	32	30
Genome length (bp)	343,806	384,670
G + C (%)	41	50
Nucleic acid identity (%)	59.29	
# of ORFs	486	592
# of tRNAs	44	56
# of ORFs with predicted functions	80	124

Comparison of the best hit protein homologs

Annotation	ORF # in		% identity
	RsoM2USA	XacN1	
Predicted ORF	ORF212	ORF239	35.76
Predicted ORF	ORF215	ORF243	36.84
DNA polymerase III alpha subunit	ORF216	ORF244	39.33
Single-stranded DNA binding protein	ORF239	ORF252	40.29
Terminase large subunit	ORF253	ORF255	40.61
Structural protein	ORF356	ORF351	44.07
Tail sheath protein	ORF359	ORF367	48.23
Baseplate wedge	ORF363	ORF323	45.54
Portal vertex protein	ORF378	ORF301	41.53
Prohead core scaffolding protein and protease	ORF379	ORF297	50.58
Hypothetical protein	ORF402	ORF267	38.45
DNA polymerase	ORF451	ORF343	36.71
DNA polymerase III epsilon subunit	ORF458	ORF223	41.18
