# Supplementary material for: Biological and Molecular Characterization of a Jumbo Bacteriophage Infecting Plant Pathogenic Ralstonia solanacearum Species Complex Strains
Source: Front Microbiol. 2021 Sep 27;12:741600. doi: 10.3389/fmicb.2021.741600 (PMC8504454; doi:10.3389/fmicb.2021.741600)
Supplement: Supplementary file 1 [file Data_Sheet_1.zip › Supplementary Table S6.PDF]

Supplementary Table S6. Codon usage and tRNA availability in jumbo phage RsoM2USA and its susceptible *R. solanacearum* strain RUN302.

Amino acid	Codon usage			Anti-codon	tRNA availability	
	codon	Cumulative frequency per 10^3			RsoM2USA	*Rs* RUN302
		RsoM2USA	*Rs* RUN302			
Ala	GCG	7.65	49.67	CGC		1
Ala	GCA	13.36	25.24	TGC	1	3
Ala	GCT	13.31	20.82	AGC		
Ala	GCC	7.29	41.33	GGC		2
Cys	TGT	16.54	9.07	ACA		
Cys	TGC	16.08	25.46	GCA	1	2
Asp	GAT	22.44	16.40	ATC		
Asp	GAC	9.3	17.10	GTC	1	3
Glu	GAG	10.37	11.71	CTC		
Glu	GAA	23.25	12.23	TTC	2	2
Phe	TTT	28.66	5.09	AAA		
Phe	TTC	27.24	12.66	GAA	2	1
Gly	GGG	4.52	15.85	CCC		1
Gly	GGA	11.88	13.19	TCC	2	1
Gly	GGT	12.26	16.66	ACC		
Gly	GGC	6.77	41.10	GCC	1	2
His	CAT	25.52	13.74	ATG		
His	CAC	11.66	17.66	GTG	1	1
Gln	CAG	15.28	22.25	CTG		
Gln	CAA	22.44	10.54	TTG	1	1
Ile	ATA	18.3	3.05	TAT		
Ile	ATT	28.62	4.88	AAT		
Ile	ATC	24.99	16.39	GAT		3
Lys	AAG	14.99	10.08	CTT	1	1
Lys	AAA	22.99	4.99	TTT	1	1
Leu	TTG	23.61	11.25	CAA	1	1
Leu	TTA	16.61	1.36	TAA	1	1

Leu	CTG	16.02	22.02	CAG		2
Leu	CTA	12.01	3.15	TAG	2	1
Leu	CTT	23.59	10.39	AAG		
Leu	CTC	11.57	11.60	GAG	1	1
Met	ATG	20.29	14.01	CAT	2	2
Asn	AAT	27.39	4.87	ATT		
Asn	AAC	17.62	9.65	GTT	2	1
Pro	CCG	8.07	37.61	CGG		1
Pro	CCA	17.43	19.93	TGG	3	1
Pro	CCT	10	14.14	AGG		
Pro	CCC	5.77	16.09	GGG		1
Gln	CAG	15.28	22.25	CTG		
Gln	CAA	22.44	10.54	TTG	1	1
Arg	AGG	8.58	14.25	CCT		1
Arg	AGA	18.02	7.56	TCT	2	1
Arg	CGG	7.83	37.58	CCG		1
Arg	CGA	11.39	26.37	TCG		
Arg	CGT	12.11	19.66	ACG		1
Arg	CGC	7.81	49.16	GCG		
Ser	AGT	13.29	5.56	ACT		
Ser	AGC	13.39	21.18	GCT	1	1
Ser	TCG	11.36	27.08	CGA	1	1
Ser	TCA	22.29	11.42	TGA	1	1
Ser	TCT	21.82	7.89	AGA		
Ser	TCC	14.85	13.42	GGA	1	1
Thr	ACG	11.81	19.47	CGT	1	1
Thr	ACA	14.69	9.01	TGT	2	1
Thr	ACT	13.56	5.42	AGT		
Thr	ACC	14.11	16.50	GGT	1	1
Val	GTG	12.42	17.69	CAC		1
Val	GTA	13.69	5.62	TAC	2	1
Val	GTT	17.78	9.73	AAC		
Val	GTC	11.66	17.39	GAC	1	1
Trp	TGG	17.1	20.19	CCA	1	1
Tyr	TAT	16.75	3.03	ATA		

Tyr	TAC	12.87	5.42	GTA	2	1
End	TGA	24.57	11.26			
End	TAG	14.22	3.49			
End	TAA	16.33	1.38			
Sup		0		CTA	1	
